# Mesenchymal Stem Cell Immunomodulation: A Novel Intervention Mechanism in Cardiovascular Disease

**DOI:** 10.3389/fcell.2021.742088

**Published:** 2022-01-12

**Authors:** Yueyao Wang, Zhongwen Qi, Zhipeng Yan, Nan Ji, Xiaoya Yang, Dongjie Gao, Leilei Hu, Hao Lv, Junping Zhang, Meng Li

**Affiliations:** ^1^ First Teaching Hospital of Tianjin University of Traditional Chinese Medicine, Tianjin, China; ^2^ National Clinical Research Center for Chinese Medicine Acupuncture and Moxibustion, Tianjin, China; ^3^ Institute of Gerontology, Xiyuan Hospital, China Academy of Chinese Medical Sciences, Beijing, China

**Keywords:** cardiovascular disease, immune regulation, inflammation, mesenchymal stem cells, immune cells

## Abstract

Mesenchymal stem cells (MSCs) are the member of multipotency stem cells, which possess the capacity for self-renewal and multi-directional differentiation, and have several characteristics, including multi-lineage differentiation potential and immune regulation, which make them a promising source for cell therapy in inflammation, immune diseases, and organ transplantation. In recent years, MSCs have been described as a novel therapeutic strategy for the treatment of cardiovascular diseases because they are potent modulators of immune system with the ability to modulating immune cell subsets, coordinating local and systemic innate and adaptive immune responses, thereby enabling the formation of a stable inflammatory microenvironment in damaged cardiac tissues. In this review, the immunoregulatory characteristics and potential mechanisms of MSCs are sorted out, the effect of these MSCs on immune cells is emphasized, and finally the application of this mechanism in the treatment of cardiovascular diseases is described to provide help for clinical application.

## 1 Introduction

Mesenchymal stem cells (MSCs) are self-sustaining stromal cells that are mainly found in bone marrow (BM), adipose tissue, fat, and umbilical cord. The multipotency of MSCs makes them an attractive therapeutic tool for organ transplantation in modern medicine (Ding et al., 2011). Currently, MSCs have been considered as a potential measure for the treatment of autoimmune and inflammatory diseases. Notably, clinical and experimental studies have demonstrated the therapeutic role of MSCs in cellular immunomodulation, organ-specific, and systemic inflammatory diseases. Recently, it has been demonstrated that MSCs can regulate the immune response of the body by regulating macrophage polarization, producing immunosuppressive molecules, and stimulating metabolites (Harrell et al., 2019a).

Cardiovascular diseases are currently the leading cause of death worldwide (Wang and Zhao. 2018). They are ischemic heart diseases that occur in the heart and its surrounding vessels, caused by lesions such as atherosclerosis, hypertension, and hyperlipidemia (Albany et al., 2019). MSCs have been widely concerned as a potential treatment for cardiovascular disease due to their potential to repair cardiac injury. Meta-analysis of animal studies has demonstrated that MSCs are safe and effective in the treatment of ischemic heart disease and have a great improvement in left ventricular fraction (LVEF). In previous studies, MSCs have been reported to repair damaged myocardium, promote myocardial regeneration, and restore normal cardiac function. They secrete various cytokines such as epidermal growth factor through the paracrine pathway for cardiac repair and regulate the expression of immune cells and related inflammatory cells to alleviate the inflammatory response after myocardial injury. Therefore, MSCs play a role in protecting cardiac function and treating cardiovascular diseases (Liu et al., 2020). This review explains the immunomodulatory effects of MSCs and their role in cardiovascular diseases, in order to provide help for the application of bone marrow stem cells in cardiovascular disease.

## 2 Overview of Mesenchymal Stem Cells

MSCs are early cells in mesoderm development and have differentiation potential, which were discovered by Alexander Friedenstein in the late 1960s (Uccelli et al., 2008; Spees et al., 2016). At present, the stem cells that can be used for research mainly include human umbilical cord mesenchymal stem cells (UCMSCs), adipose-derived mesenchymal stem cells (ADMSCs), BM mesenchymal stem cells (BMMSCs), and placenta-derived mesenchymal stem cells (PlaMSCs) (Li et al., 2015a). MSCs have high proliferation and self-renewal ability and can differentiate into neural cells, cardiomyocytes, blood cells, and other cells at different development stages and in different environments (He et al., 2013). MSCs are capable of repairing tissue damage through their differentiation function, and can also express a variety of characteristic immunophenotypes, such as CD105\CD105\CD90\CD44\CD71 (J and Debabrata. 2017).

MSCs secrete immunomodulatory factors, cytokines, growth factors, extracellular vesicles (EV) and other bioactive factors such as, which anti-apoptotic, anti-fibrotic, antioxidant and immunomodulatory effects and regulate a series of physiological processes. MSCs have been shown to secrete cytokines such as Chemokine C-C motif ligand 2 (CCL-2), Chemokine C-C motif ligand 5 (CCL-5), Insulin-like growth factor-1 (IGF-1), Interleukin-6 (IL-6), and Vascular endothelial growth factor (VEGF), which are involved in cell and tissue development, differentiation, and death (Wang et al., 2019). MSCs perform immune regulation by secreting cytokines such as IL-6, Interleukin-10 (IL-10), prostaglandin E2 (PGE2) and transforming growth factor-beta (TGF-β) (Lopatina et al., 2019), and can also participatein intercellular communication by secreting exosomes with immunomodulatory characteristics, thereby facilitating the immune system to recognize and eliminate antigenic foreign bodies (Lee et al., 2012). The chemokines of MSCs are mainly CXC chemokine receptor 3 (CXCR3) and CC chemokine receptor 5 (CCR5) ligands, as well as CXC chemokine ligand 9 (CXCR9), CXC chemokine ligand 10 (CXCR10), and CXC chemokine ligand 11(CXCR11). They are well-known immune cell chemotactic agents capable of modulating immune cells, such as T lymphocytes (T cells) (Shi et al., 2018a). MSCs regulate the immune response by regulating the activity of T cells and B -lymphocytes (B cells), thus inhibiting cell apoptosis. They interact with the immune system to induce the immunosuppressive activity of many immune cells, including lymphocytes, antigen-presenting cells, and natural killer cells (NK cells). In recent years, MSCs have gained much attention due to their potential application value in the treatment of autoimmune diseases (Table 1).

**TABLE 1:** Summary of cytokines involved in immunomodulation of MSCs.

Cytokines	Roles in MSCs Immunomodulation	References
IL-2	Inhibition of NK cells proliferation and differentiation	Spaggiari et al. (2006)
IL-6	Promotion of B cells proliferation and differentiation	Qin et al. (2015)
	Inhibition of DCs differentiation	Consentius et al. (2015)
IL-10	Promotion of B cells proliferation and increase in the number of B-reg cells	Gupte et al. (2017)
PGE2	Inhibition of DCs differentiation. Reduction of T cells expression. Induction of M2 phenotype of macrophages	Spaggiari et al. (2009), Yang et al. (2018), Jin et al. (2019)
TGF-β	Inhibition of DCs proliferation and activation	De Miguel et al. (2012)
HGF	Regulation of CD4^+^ T cells, and Th1 and Th2 cells	Özgül Özdemir et al. (2021)
CCL-2	Improvement of IFN-γ production *via* NK cells	Cui et al. (2016)
IFN-α	Inhibition of macrophages proliferation	Shou et al. (2016)

Abbreviations: IL-2, Interleukin-2; DCs, Dendritic cells; IFN-γ, Interferon γ; CCL-2, Chemokine C-C motif ligand 2; IFN-α, Interferonα.

## 3 Modulation of Different Immune Cells by Mesenchymal Stem Cells

### 3.1 Macrophages

Macrophages are essential components of the human immune system, which can perform different functions in the body’s immune response, such as regulating apoptosis, phagocytosing of pathogens, remodeling the extracellular matrix, activating other immune cells, and so on. Under various stimuli, macrophages can polarize into two phenotypes, M1 and M2 (Wynn et al., 2013; Koelwyn et al., 2018). Among the two phenotypes of macrophages, classical M1-polarized macrophages can promote the secretion of pro-inflammatory factors, promote the antibacterial reaction, and alternative M2-polarized macrophages [activated by Interleukin-4 (IL-4) and Interleukin-13 (IL-13)], which are generally considered to have the immunosuppressive effect (Wang et al., 2016a). MSCs inhibit macrophages activation and transform the M1 phenotype to the M2 phenotype.

#### 3.1.1 Regulation by Related Proteins and Inflammatory Factors

BMMSCs injected into the sulfur mustard-induced acute lung injury mouse model, the proportion of anti-inflammatory M2 macrophages was substantially increased, whereas that of M1 macrophages was decreased in BMMSCs-treated mice compared with the sulfur mustard group, and the expression of Toll-like receptor 4 (TLR4) greatly increased, that indicated BMMSCs can inhibit the differentiation of macrophages into pro-inflammatory M1 macrophages through the TRL4 signaling pathway and promote the differentiation of macrophages to anti-inflammatory M2 macrophages (Feng et al., 2019). Yap protein in the Hippo pathway can regulate the inflammatory response. MSCs control NLRP3 inflammasome assembly by activating the Hippo pathway in macrophages and regulating the interaction between yap and *β*-catenin (Li et al., 2019).

On the other hand, the immunomodulatory effect of MSCs can be achieved by secreting inflammatory factors. TGF-β secreted by MSCs activates the Akt/Forkhead box transcription factor O1 (FoxO1) pathway in mice macrophages with high body oxygen levels, alveolar fibrosis, and pulmonary vascular remodeling (Liu et al., 2019). Inflammatory chemokines CCL2 and CXC ligand 2 (CXCL2) secreted by BMMSCs play an essential role in polarizing mouse peritoneal macrophages into the IL-10 phenotype (Giri et al., 2020).

#### 3.1.2 Regulation by Secreted Exosomes

MSCs secrete vesicles with a diameter of 40–100 nm, which are called exosomes. Exosomes contain a large amount of biologically active substances, like cytokines, proteins, miRNAs, DNA, and other biologically active substances. Exosomes from BMMSCs were extracted, some of them were treated with lipopolysaccharide (LPS), the results indicated that both the normal MSCs exosomes and the LPS-treated MSCs exosomes considerably reduced the positive rate of the M1 macrophages protein marker CD11 and increased the positive rate of the M2 macrophages protein marker CD206. Exosomes derived from MSCs are able to regulate macrophages polarization by inhibiting the Nuclear Factor Kappa-B (NF-κB) pathway and activating the Serine threonine-specific protein kinase (Akt1/Akt2) pathway, thereby reducing post-infarction inflammation and cardiomyocytes apoptosis (Xu et al., 2019). In the steroid-resistant mouse model, Bing Dong and his colleagues found that intratracheal administration of exosomes treatment reduced inflammation and reduced M1 macrophages, and promoted M2 macrophages. This effect could achieve by inhibiting the TNF receptor-associated factor 1 (TRAF1) (Dong et al., 2021). In this way, MSCs promote the polarization of macrophages from a pro-inflammatory M1 phenotype to an anti-inflammatory M2 phenotype and regulate through related proteins, exosomes, and inflammatory factors.

### 3.2 Dendritic Cells

Dendritic cells are individual antigen-presenting cells. They are heterogeneous population of leukocytes with different subpopulations, which are responsible for driving specific immune responses and initiating and modulating adaptive immune responses (Lee and Radford. 2019).

#### 3.2.1 Regulation of the Proliferation, Maturation, and Differentiation of Dendritic Cells

MSCs are capable of inhibiting the proliferation, maturation, and differentiation of DCs, thus exerting immunomodulatory and immunosuppressive effects (De Miguel et al., 2012). MSCs and DCs derived from mouse bone marrow were co-cultured, and the results showed that MSCs derived exosomes were able to reduce the expression of surface markers, and inhibit the maturation of DCs treated with LPS (Zhang et al., 2017). TGF-β1 expressing lentiviruses were used for MSCs transduction, and then these MSCs were co-cultured with T cells and DCs, and the results showed that MSCs derived exosomes were able to inhibit the maturation of DCs through TGF-β and had an immunosuppressive effect (Daneshmandi et al., 2017).

DCs have numerous phenotypes, including major histocompatibility complex I (MHC-I) and major histocompatibility complex II (MHC-II) as well as CD370, CD207, CD205, CD1a, CD11c, CD11b, CD83, and CD40 (Clark et al., 2019). Hypoxia-inducible factor (HIF) exert significant biological effects such as angiogenesis and cell survival. Transduction of MSCs with GFP-HIF-1α lentiviral vector (HIF-MSCs) followed by co-culture with DCs revealed that HIF-MSCs significantly decreased DCs differentiation and increase resistance to NK cell lysis (Martinez et al., 2017).

#### 3.2.2 Regulation of the Interaction Between Dendritic Cells and Lymphocytes

**FIGURE 1:**
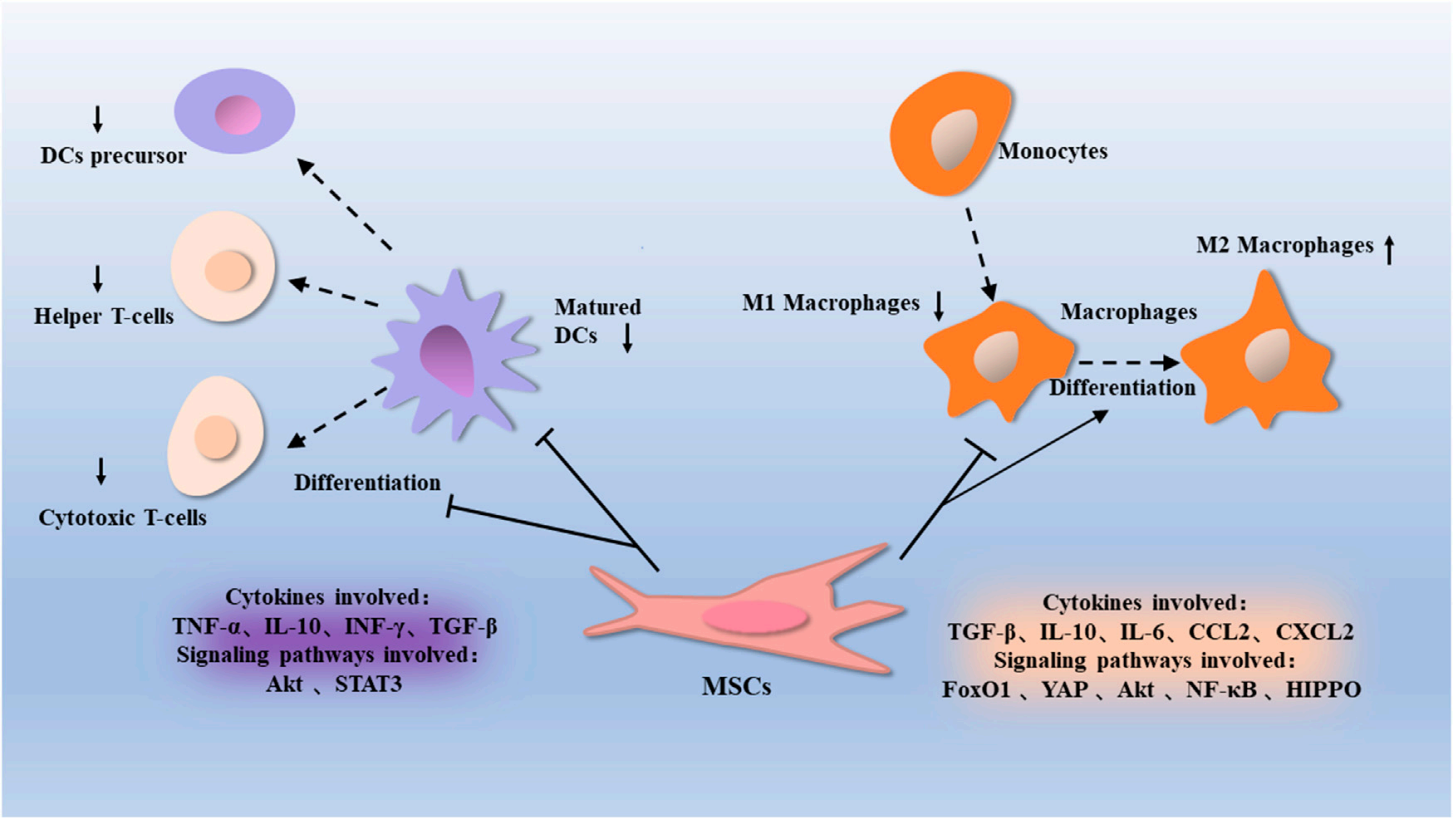
Macrophages are a type of immune cells that differentiate from differentiated monocytes derived from MSCs. MSCs inhibit macrophages activation through proteins such as Akt, FoxO1, NF-κB, and Hippo, can promote the conversion of macrophages from a pro-inflammatory M1 phenotype to an anti-inflammatory M2 phenotype. This is associated with factors such as TGF-β, IL-10, IL-6, CCL2, and CXCL2. Dendritic cells are differentiated from dendritic cell precursors and regulate the conversion of T cells to helper T cells and cytotoxic T cells. MSCs inhibit the proliferation of dendritic cells through proteins such as Akt and STAT3, thus affecting their regulatory T-cell function, which is associated with factors such as TNF-α, IL-10, INF γ, and TGF-β.

Dendritic cells can initiate T cells immunoregulation, and they regulate T cells differentiation toward Th1, Th2, Th17, or Tregs subpopulations. DCs also indirectly regulate T cells subpopulation differentiation by activating intrinsic lymphocytes to produce regulatory cytokines (Briseño et al., 2014). Immature dendritic cells (Im-DCs) and LPS-treated DCs were co-cultured with MSCs for 48 h. The expression of CD11c, CD80, CD86, and IL-6, Tumor necrosis factor *α* (TNF-α), and IFN-γ was decreased, while CD11b, IL-10, and TGF-β expression was largely increased and stimulated splenocytes to produce markers of Tregs (FOXP3, CD4, and CD25). Thus, MSCs can induce the transformation of immature DC phenotypes to regulatory dendritic cells (r-DCs), and MSCs which secrete anti-inflammatory cytokines (IL-10 and TGF-β) play a similar role to r-DCs, leading to the activation of Tregs (Jo et al., 2018). Tumor MSCs were able to inhibit DCs’ expression of cysteine through Signal transduction and activation protein 3 (STAT3), thereby preventing DCs from promoting the expansion of naive CD4^+^ and CD8^+^ T cells (Ghosh et al., 2016). So, MSCs regulate immune regulation by regulating the differentiation and maturation of DCs and the interaction between DCs and lymphocytes (Figure 1).

### 3.3 T Lymphocytes

MSCs are allowed to mediate immune responses by regulating T cells’ activity. T cells are immune cells that originate from the BM and differentiate and mature in the thymus gland. T cells are essential for the body’s immune response, maintenance of homeostasis, maintenance of immune memory, and recognition of pathogens (Kumar et al., 2018).

#### 3.3.1 Regulation of the Activation and Proliferation of T Lymphocytes

Studies have shown that MSCs modulate immunity through T cells. Cytochalasin B-induced membrane vesicles (CIMVs) are an innovative therapeutic tool, MSCs-derived cytochalasin B membrane vesicles (CIMVs-MSCs) significantly inhibit the activation of helper T cells and cytotoxic T cells. *In vitro* studies have shown that CIMVs-MSCs cannot induce immune responses in mice, suggesting that CIMVs-MSCs have an immunosuppressive effect (Gomzikova et al., 2020). Intervention with exosomes given to in a mouse model of primary sclerosing cholecystitis (PSC) revealed that exosomes were able to reduce the extent of liver fibrosis and decrease the proliferation of T cells in the liver by inhibiting the NF-κB signaling pathway (Angioni et al., 2020).

#### 3.3.2 Regulation the Differentiation and Phenotypic Transformation of T Lymphocytes

IFN-γ is a member of the type II interferon, which is widely involved in the immune response. BMMSCs were found to promote the phenotypic transformation of T cells. BMMSCs were administered to alum-treated mice and were found to cause Th2-mediated eosinophilic pneumonia. BMMSCs can promote the Th1 phenotype of antigen-specific CD4 +cells and inhibit Th2-mediated allergic airway inflammation through an IFN-γ dependent process (Goodwin et al., 2011). Co-cultured MSCs with CD4^+^ cells and found MSCs downregulate Th1/Th17 immune responses in a PGE2-dependent manner (Zhou et al., 2020). Tregs can act through cell-to-cell contact, modulate inhibitory cytokines, such as IL-10, and modulate body immunity by interacting with antigen-presenting cells. Accordingly, they play an essential role in maintaining immune homeostasis (Wing et al., 2019). MSCs are able to induce Tregs production (Zhang et al., 2018). Milad Riazifar and his colleagues used the autoimmune encephalomyelitis (EAE) mouse model to assess the therapeutic effects of exosomes secreted by BMMSCs in multiple sclerosis, and found that the number of CD4+, CD25+, and Forkhead box P3 (FOXP3)^+^ Tregs on the spinal cord of mice upregulate after intravenous injection of IFN-γ (IFN-γ-Exo)-stimulated MSCs-derived exosomes (Riazifar et al., 2019). In conclusion, MSCs intervene in the activation, proliferation, and phenotypic transformation of T cells, as well as to increase the level of Tregs, thus exerting an immunomodulatory effect.

### 3.4 B Lymphocytes

B cells are derived from stem cells of BM, mainly located in human lymph nodes and spleen. B cells can involve in regulating the immune response, which is related to their ability to produce antibodies. Mature B cells may become activated B cells after being stimulated by antigen, and then differentiate into plasma cells to secrete and synthesize antibodies, to perform humoral immunity (Mauri and Bosma. 2012; Zhang et al., 2012).

#### 3.4.1 Regulation of the Proliferation of B Lymphocytes

MSCs are capable of affecting the activity of B cells by the immunomodulators such as cytokines, chemokines, and growth factors. In one study, it was found that MSCs from human term placental amniotic membrane (hAMSCs) and conditioned medium-derived mesenchymal stem cells (CM-hAMSCs) generated from their culture had an effect on B cells proliferation and differentiation, hAMSCs and CM-hAMSCs both strongly inhibited the proliferation of CpG-activated B cells. In addition, CM-hAMSCs blocked B-cell differentiation, resulting in an increased proportion of mature B cells and reduced formation of antibody-secreting cells (Magatti et al., 2020). It has been shown that hAMSCs have immunosuppressive effects on B cells and constitutively expresses high levels of the immunosuppressive ligand programmed cell death 1 ligand 1 (PD-L1) in response to IFN-γ (Wang et al., 2016b).

#### 3.4.2 Regulation of the Differentiation and Phenotypic Transformation of B Lymphocytes

The immunomodulatory effect of MSCs on B cells reflect in the inhibition of their differentiation and phenotypic transformation. In a mouse model of colitis, intraperitoneal injection of BMMSCs could regulate the immunomodulatory effects of B cells by upregulating IL-10 expression, induce a regulatory B-cell (Breg) population characterized by CD23 and CD43 phenotypic markers, increase the number of CD23^+^, CD43^+^, and Breg cells, reduce the clinical and pathological severity of colitis in mice (Chen et al., 2019a). Researchers studied the interactions of BM-MSCs and placental MSCs (P-MSCs) in the mouse model, found that P-MSCs could inhibit the proliferation and further differentiation of B cells (Lee et al., 2021).

The immunomodulatory effects of MSCs on B cells have been studied in many clinical therapies. In a clinical study on chronic graft-versus-host disease, 38 patients treated with MSCs showed a remarkable increase in the number of CD27^+^ and memory B cells, while plasma B-cell activating factor (BAFF) levels decreased and BAFF-R (BAFF receptor) expression increased on peripheral B cells (Peng et al., 2014).

Although MSCs play a substantial immunosuppressive role for B cells by inhibiting the proliferation of B cells through inflammatory factors such as IFN-γ and TNF-α. Remarkably, the regulatory role of MSCs on B cells is still under investigation, some studies have found that MSCs have a promotive effect on B-cells proliferation, and BMMSCs of B cells chronic lymphocytic leukemia (B-CCL) patient origin were shown to greatly inhibit B cells proliferation and immunoglobulin G (IgG) secretion compared to normal MSCs (Pontikoglou et al., 2013). Co-cultures of MSCs with lymphocytes showed that MSCs stimulate antibody secretion from B cells, and whether antibody secretion from B cells was inhibited or promoted was determined by the dose of MSCs (Rasmusson et al., 2007). However, in some studies, co-culture of LPS-stimulated B cells with MSCs revealed that the proliferation and differentiation of B cells can be inhibited (Asari et al., 2009). Adipogenic-differentiated MSCs (Adi-MSCs) can stimulate B cells proliferation and activation, and promote the secretion of BAFF in the presence of anti-CD3 and anti-Mu-chain treatment (Wang et al., 2011).

### 3.5 Natural Killer Cells

NK cells, also known as natural killer cells, are critical immune cells produced by the body, and they are involved in hypersensitivity reactions. NK cells have many receptors that receptors tightly regulate the activity of NK cells, enabling them to distinguish between “normal” and “dangerous” cells (Morvan and Lanier. 2016; Hodgins et al., 2019).

#### 3.5.1 By Regulating the Proliferation and Degranulation Effects of Natural Killer Cells

MSCs could be immunomodulated by NK cells. It has shown that BMMSCs have the ability to promote the degranulation effect of NK cells, which can occur when NK cells are near target cells due to components such as perforin and granzyme, and degranulation is associated with the killing activity of NK cells, which directly kill target cells and exert innate immunity (Bradley et al., 2018). NK cells of peripheral blood mononuclear cells originate from healthy donors incubated with SKO-007 (J3) cells (Human myeloma cells), some of the cells were untreated, and some were incubated with BMSC-CM for 72 h, as target cells for degranulation assay. The results showed that CD107a expression was substantially elevated in the combined BMMSC incubation group, indicated that the degranulation of NK cells was enhanced (Mekhloufi et al., 2020). MSCs inhibit NK cells by inhibiting IL-2 too. In a study by Grazia Maria Spaggiari and his colleagues, they explored the result of possible interactions between NK cells and MSCs, and it was found that MSCs not only inhibited the proliferation of NK cells but also prevented the induction of effector functions, and they also observed that MSCs strongly inhibit interleukin-2 (IL-2)-induced NK-cell proliferation (Spaggiari et al., 2008).

#### 3.5.2 Regulation of Activation and Phenotype of Natural Killer Cells

BMMSCs are able to modulate the immune activity of hepatic NK cells. In the Con A mouse model of liver injury, BMMSCs intervention was performed and BMMSCs transplantation was found to reduce cytotoxic substances and activation of r NK cells in the mouse liver (Qingqing et al., 2014). Studies have shown that activation of MSCs between tissues modulate the immune function of NK cells as well, and MSCs could secrete type I interferon to enhance NK cells effector function, while at subsequent time points, TGF-β and IL-6 could limit NK cells effector function and terminate the inflammatory response by inducing a regulatory senescence-like NK cells phenotype (Petri et al., 2017). Additionally, NK cells and MSCs have a bidirectional role in regulating and influencing NK cells activity. Pre-activated MSCs were found to notably inhibit the expression of activation markers in hepatic NK cells after co-transplantation with pancreatic islets to regulate NK cells activity (Ishida et al., 2019). Therefore, MSCs can interfere with the degranulation of NK cells and have an inhibitory effect on the proliferative killing activity of NK cells, which is associated with cytokines such as IL-2 and IFN-γ.

**FIGURE 2:**
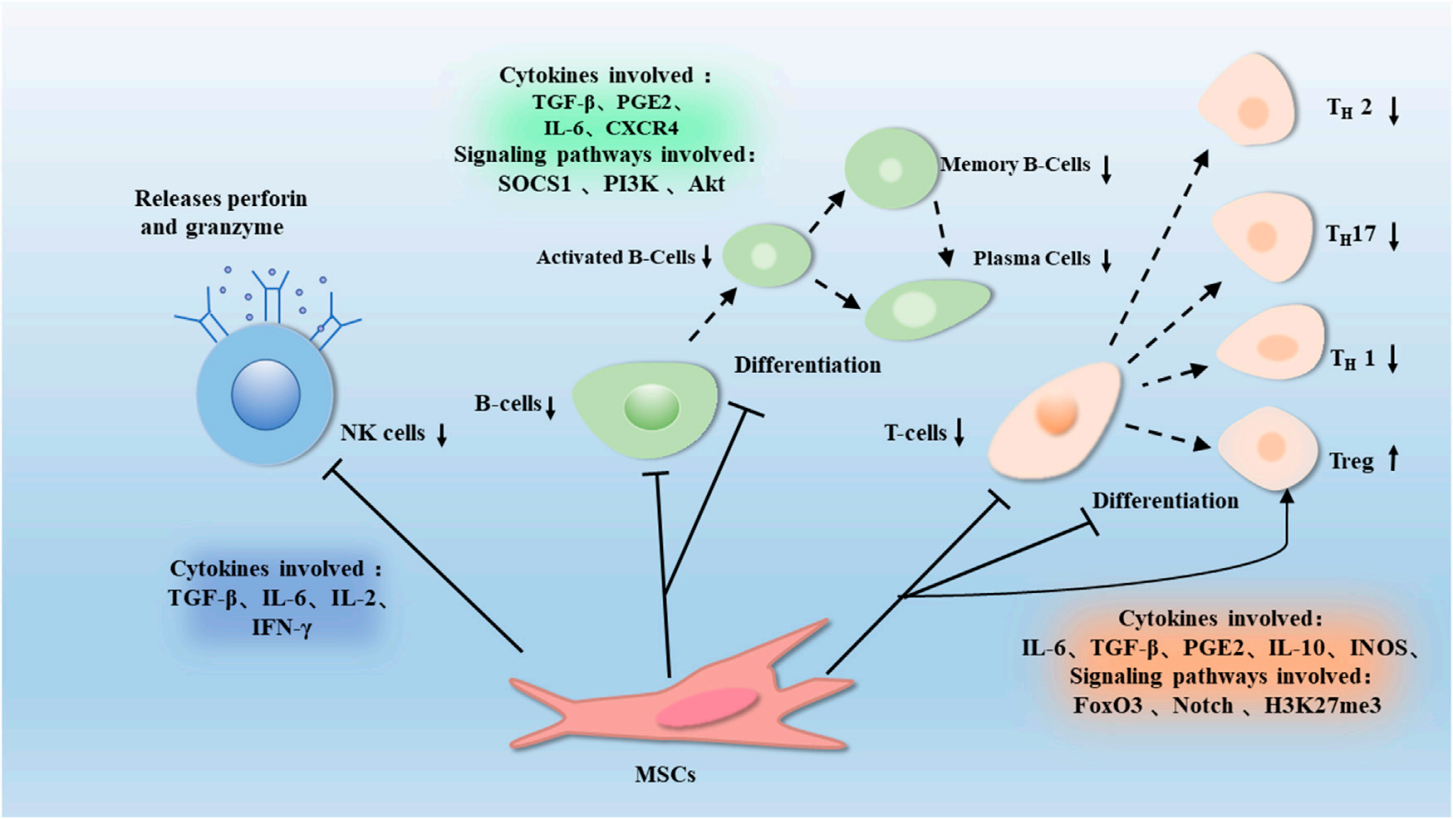
T cells can divide into different subpopulations during immune regulation, such as Th1, Th7, Th2, and Tregs. MSCs could inhibit suppression of T cells proliferation and differentiation, and induce Tregs through proteins such as H3K27me3, Notch and FOXP3. This effect is associated with the release of Soluble factors such as TGF-β, IL-10, PGE-2, IL-6, and IDO. MSCs gradually differentiate into B cells, which can differentiate and proliferate into plasma cells. MSCs inhibit the proliferation of B cells and antibody production through proteins such as Socs1, PI3K, and Akt, which are associated with factors such as TGF-β, PGE2, IL-6, and CXCR4. MSCs also promote the release of perforin and granzyme from NK cells, thus promoting the degranulation of NK cells, which is associated with factors such as TGF-β, IL-6, IL-2, and IFN-γ.

We have described the way MSCs regulate immunity between different cells. We know that endothelial cells, macrophages, neutrophils, lymphocytes, monocytes, etc. accompany different roles in cardiovascular diseases and whether MSCs can further play a therapeutic role by influencing the immune metabolism of these cells. Therefore, we will focus our discussion on the therapeutic role of MSCs in a variety of cardiovascular diseases (Figure 2).

## 4 Immunomodulation of MSCs in Cardiovascular Disease

### 4.1 Myocardial Infarction

Myocardial infarction (MI) is a disease caused by severe narrowing of the coronary arteries and a dramatic reduction or interruption of the blood supply, which leads to severe acute ischemia in the myocardium and ischemic necrosis. Myocardial ischemia over a long period of time can lead to myocardial cell death, severe angina pectoris, arrhythmias, and other symptoms, which could be life-threatening in severe cases.

MSCs are able to reduce cardiac dysfunction through immunomodulatory effects. The reason why MSCs can treat heart disease may be due to promoting the increase in the number of M2 macrophages. In the mice MI model, MSCs were injected into mice with BM-derived macrophages and found that MSCs could reduce M1 phenotypic markers (IL-6, IL-1β) and increase M2 phenotypic markers (IL-10, IL-4), and facilitated the recovery of cardiac function (Cho et al., 2014). Interleukin-33 (IL-33) is the tissue-derived nuclear cytokine from the IL-1 family, its function is to release alarm signals in case of cell damage or tissue damage. It targets mast cells, Tregs, NK cells, neutrophils, and macrophages in the body, making it a vital important immunomodulatory molecule (Cayrol and Girard. 2018). MSCs transfected with IL-33 were co-cultured with T cells and macrophages, and the proliferation of T cells and polarization of macrophages were observed. The experiments showed that IL33-MSCs decreased T cells proliferation, and enhanced the polarization of M1 phenotype to M2 phenotype, and the myocardial fibrosis, inflammation, and cardiac function recovered better with IL33-MSCs (Chen et al., 2019b).

In addition to IL-33, IL-10 is also an important immunomodulatory factor. IL-10 is a multipotent cytokine with properties that prevents host damage by limiting the immune response of pathogens and exerts immunosuppressive or immunostimulatory effects in various cells (Saraiva and O'Garra. 2010). MSCs are able to perform immunomodulatory functions by regulating IL-10, IL-10-transfected MSCs were injected into MI mice and found that IL-10-transfected MSCs enhanced cell viability and increased IL-10 secretion, the infarct size, myocardial injury and apoptosis were reduced, and systemic and local inflammation were reduced (Meng et al., 2018).

MSCs play a role in tissue repair of cardiovascular diseases. Studies have shown that after MI, MSCs can differentiate into endothelial cells or cardiomyocytes and exert their immunomodulatory effects, such as alleviating tissue damage and promoting tissue repair by inhibiting neutrophil infiltration. Alternatively, tissue repair is achieved by altering the distribution of effective immune cells and recruiting M2 macrophages to the vicinity of damaged tissue. In the murine model of MI, injection of MSCs significantly decreased the M1 phenotype of macrophages, decreased the expression of Interleukin-1β (IL-1β) and IL-6, increased the expression IL-10, and increased the alternating activation of monocytes/macrophages. MSCs may repair the myocardium through IL-10-mediated infiltration of pro-inflammatory macrophages to anti-inflammatory macrophages at the site of infarction (Dayan et al., 2011). MSCs were injected in the porcine model of heart failure combined with MI and were found to exhibit enhanced viability and promote vascular regeneration by activating Tregs and reducing inflammatory cells (Liao et al., 2019).

However, single MSCs therapy tends to have low delivery rates. In the rat model of MI, it was found that combined delivery of MSCs and MSCs-derived exosomes were able to achieve more efficient anti-inflammatory and vascular tissue repair than MSCs therapy by decreasing the expression of inflammatory factors such as IL-6 and TNF-α and increasing the expression of Recombinant Stromal Cell-Derived Factor 1 (SDF-1) (Huang et al., 2019).

MSCs and their derived materials are emerging in the treatment of MI as well. Exosomes of porcine cardiac adipose tissue-derived MSCs (cATMSC) loaded onto a three-dimensional scaffold designed from decellularized heart tissue and injected into pigs after MI, found to reduce macrophage and T cell infiltration in the damaged myocardium and increase vascular density and tissue remodeling (Monguió-Tortajada et al., 2021). Researchers coated the cell membrane of MSCs on PLGA particles loaded with MSCs secretions to create a particle called synMSCs and injected it into mice with acute myocardial infarction (AMI), and found that synMSCs-treated mice were able to express MHC I molecules that allowed them to avoid allogeneic recognition by the immune system, thereby modulating the body’s innate immune response and enhancing their attachment to cardiomyocytes and promoting their survival by secreting adhesion factors (such as SDF1) (Luo et al., 2017). Vineeta Sharma and his colleagues found that 5-Aza loaded protein nanoparticle with MSCs encapsulated hydrogels may support *in vitro* MSC proliferation, migration and angiogenesis in rat MI model, and the hydrogels could alleviate ventricular remodeling after myocardial infarction by secreting immunoregulatory factors (Sharma et al., 2021). The addition of MSCs -derived exosomes to alginate hydrogel revealed that the delivery of exosomes incorporated in alginate hydrogel (sEVs-Gel) increased residence time in the heart, and promoted the polarization of macrophages from M1 phenotype to M2 phenotype more than exosomes treatment alone, promoted scar tissue repair and vascular regeneration (Lv et al., 2019). Some clinical treatments with MSCs have shown encouraging results. For example, in a clinical study of patients with chronic ischemic heart disease, patients were found to reduce myocardial infarct size, increase LVEF and promote recovery of cardiac function after intravenous administration of MSCs (Kanelidis et al., 2017).

### 4.2 Heart Failure

Heart failure is a progressive disease, the syndrome that occurs when the cardiovascular diseases progress to a more severe stage. It is usually caused by structural and functional changes in the myocardium due to organic or functional lesions of the heart, which impairs the filling degree and ejection fraction capacity of the ventricles. Furthermore, immune activation and inflammatory responses play an essential role in the course of heart failure.

The therapeutic effect of MSCs on heart failure is mainly based on their ability to regulate the body’s innate and adaptive immunity. Peripheral blood mononuclear cells were isolated from fresh blood samples of patients with end-stage heart failure and co-cultured with MSCs derivatives. The results showed that MSCs had a strong immunosuppressive ability and inhibited lymphocyte proliferation and antibody production *in vitro*, and plasma cells from patients with end-stage heart failure had high IgG3 production, *in vitro* experiments showed that MSCs could inhibit IgG3 production, thereby preventing ventricular remodeling after MI and slowing the progression of heart failure (van den Hoogen et al., 2019). CD4 is generally expressed on the surface of human T cells, while Th1 cells in CD4 have a role in humoral immunity (Loo et al., 2018). CD4/CD8 is an important indicator of immune regulation (Overgaard et al., 2015). Co-transplantation of MSCs and pluripotent stem cell-derived cardiomyocytes into mice revealed that MSCs directly affected activated lymphocytes through cell-to-cell contact, thereby decreasing the CD4/CD8 ratio and the proportion of Th1-positive cells among CD4-positive cells and the secretion of various inflammation-related cytokines. Moreover, this pathway could increase the number of cardiomyocytes, enhance myocardial contraction (Yoshida et al., 2020). Denise Philipp and his colleagues studied the effects of bone marrow-derived preconditioned MSCs on hypertrophy-induced pluripotent stem cell-derived cardiomyocytes (iPS-CM), and found that MSCs could participate in immune regulation by secreting cytokines such as IFN-γ and IL-1β, and trigger regression of hypertrophy in iPS-CM in a VEGF-dependent manner, so MSCs therapy may inhibit cardiac hypertrophy (Philipp et al., 2021).

Some clinical studies have shown that MSCs is a new target for the treatment of heart failure. After MSCs injected into patients with chronic heart failure, a momentous decrease in CD4-positive and NK cells was found, a large improvement in left ventricular end systolic volume (LVESV) and LVEF was observed. In this study, MSC therapy was safe, and caused immunomodulatory effects (Butler et al., 2017). Intravenous infusion of UC MSCs in patients with chronic heart failure revealed that UC MSCs could participate in the regulation of adaptive immunity and myocardial remodeling in the body by secreting hepatocyte growth factor (HGF), inhibit T-cell proliferation, and reduce the proliferation of Th 1, Th 2, and cytotoxic T cells (Bartolucci et al., 2017).

**FIGURE 3:**
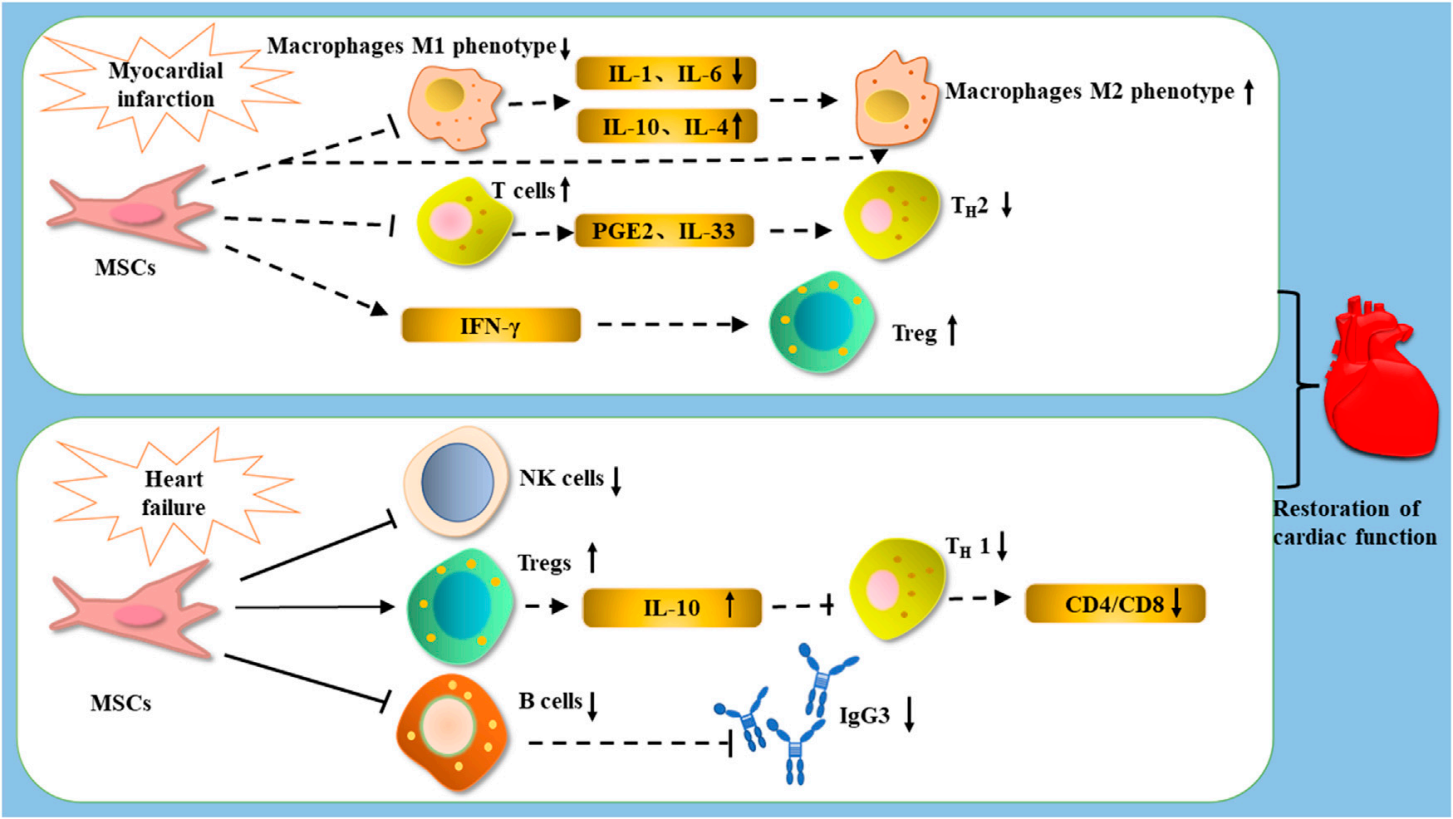
After the occurrence of MI, treated with MSCs, MSCs improve cardiac function by decreasing the expression of IL-1β and IL-6, increasing the expression of IL-10 and IL-4, and promoting the polarization of macrophages from M1 phenotype to M2 phenotype; they can improve cardiac function by promoting the expression of Tregs to enhance cardiac function, as well as inhibiting T cells and influencing the differentiation of Th1 cells through PGE2 and IL-33. After the occurrence of heart failure, treatment with MSCs can restore cardiac function by reducing the number of NK cells, inhibiting the proliferation of B cells and the production of the antibody IGg3, as well as increasing the number of Tregs and reducing the CD4/CD8 ratio by reducing Th1 cells through IL-10.

MSCs derived materials are also a new target for the treatment of heart failure in recent years. Human amnion-derived stem cells (hAM-MSCs) dressing is a novel MSC-derived material with good potential for clinical application. After administration of hAM-MSCs dressing to mice with ischemic cardiomyopathy, it was found that the dressing could improve myocardial remodeling by promoting the secondary release of paracrine factors from endogenous cells and promoting the polarization of the M1 phenotype to the M2 phenotype of macrophages. So, hAM-MSCs dressing can slow down the development of heart failure (Fields et al., 2021). Pura-Matrix hydrogel with MSCs applied to mouse epicardium after MI, found that it could inhibit the development of inflammation, promote the formation of myocardial capillary, reduce interstitial fibrosis, improve cardiac function and alleviate the progression of heart failure by secreting cytokines such as IL-10 (Ichihara et al., 2018). Researchers seeded MSCs and macrophages into a polycaprolactone scaffold and filled it with MSCs-derived exosomes, found that this novel MSCs-derived material increased the wound healing properties and type I collagen of MSCs and improved the M2 phenotype of macrophages, thus this exosome-rich bio scaffold is an effective strategy to restore contractility of myocardial scars (Chachques et al., 2021). Both MSCs and their derived materials can play a role in the treatment of heart failure by secreting immune factors and regulating immune cells (Figure 3).

### 4.3 Atherosclerosis

Atherosclerosis is a chronic vascular inflammatory disease. That is the cause of coronary heart disease. Atherosclerotic lesions are associated with macrophages, T cells and other immune response cells (Hansson and Hermansson. 2011).

Because of their immunomodulatory and tissue regenerative capabilities, MSCs are well-positioned to treat atherosclerosis, And the therapeutic effect of MSCs on atherosclerosis is mainly achieved by regulating macrophages. As we all known, abnormal lipid metabolism can lead to endothelial dysfunction and the secretion of adhesion factors, leading to the recruitment of macrophages to form foam cells, promoting atherosclerotic plaque formation. Macrophages secrete anti-inflammatory factors, such as IL-10, TGF-β, and promote blood vessel regeneration and tissue remodeling and repair (Adutler-Lieber et al., 2013). Therefore, the treatment of atherosclerosis with MSCs is mainly completed by regulating macrophages, and some immune factors also play a role in this process. For example, IL-6 is produced in response to infection and tissue injury and contributes to host defense by stimulating the acute phase response, hematopoiesis, and immune response. Persistent dysregulation of IL-6 synthesis plays a pathological role in chronic inflammation and autoimmunity (Tanaka et al., 2014). Human-induced pluripotent stem cells (iPSC-MSCs) were intravenously administered to ApoE ^−/−^mice on an HFD for 12 weeks, and iPSC-MSCs were found to notably reduce plaque size and make the macrophages in the plaques lower than those in the controls. Furthermore, iPSC-MSCs reduced inflammation by reducing serum levels of inflammatory cytokines, such as TNF-α and IL-6, thereby treating atherosclerosis (Shi et al., 2018b). Skin is an ideal source of MSCs supply, and in the ApoE ^−/−^ mice model, skin-derived MSCs(S-MSCs) were found to migrate into atherosclerotic plaques and selectively adhere in the vicinity of macrophages. Furthermore, it reduced the release of the pro-inflammatory cytokine TNF-α and increased the expression of anti-inflammatory factor IL-10 in atherosclerotic plaques, resulted in the regulation of macrophages function and inhibition of atheromatous plaque formation (Li et al., 2015b). MSCs-derived exosomes are considered a new target for the treatment of atherosclerosis. MSCs-derived exosomes injected into ApoE^−/−^ mice and MSCs-derived exosomes were found to promote polarization of the macrophages M2 phenotype and reduce plaque size and macrophages infiltration *via* miRNA-21a-5p (Ma et al., 2021).

In addition to macrophages, Treg and T cells are also able to play a role in the treatment of MSC. TGF-β1 could produce an immune response when it is activated, and Tregs can suppress proximal immune cells by activating Glycoprotein -A repetitions predominant (GARP) -mediated activation of potential TGF-β1 acting on their surface with integrin αVβ8. Treatment of atherosclerotic mice with MSCs revealed increased secretion of anti-inflammatory cytokines such as TGF-β1 and IL-10 and TGF-β1 could be involved in MSC-mediated changes in the number of CD4^+^CD25^+^ forkhead box P3 (FOXP3) ^+^Tregs and NK cells, and reduce their proliferation, this resulted in the regulation of host adaptive immunity (Li et al., 2017; Liénart et al., 2018). Mice were injected with MSCs after low-density lipoprotein (LDL) knockout and fed a high-fat diet (HFD), which induced atherosclerosis, it was found that the number of T cells reduced after MSCs treatment, the serum CCL2 level decreased, the lesion in the aortic root reduced, the number of macrophages at the lesion site decreased, and serum cholesterol reduced. It was shown that treatment with MSCs greatly reduced dyslipidemia and treated atherosclerosis in mice (Wang et al., 2015).

**FIGURE 4:**
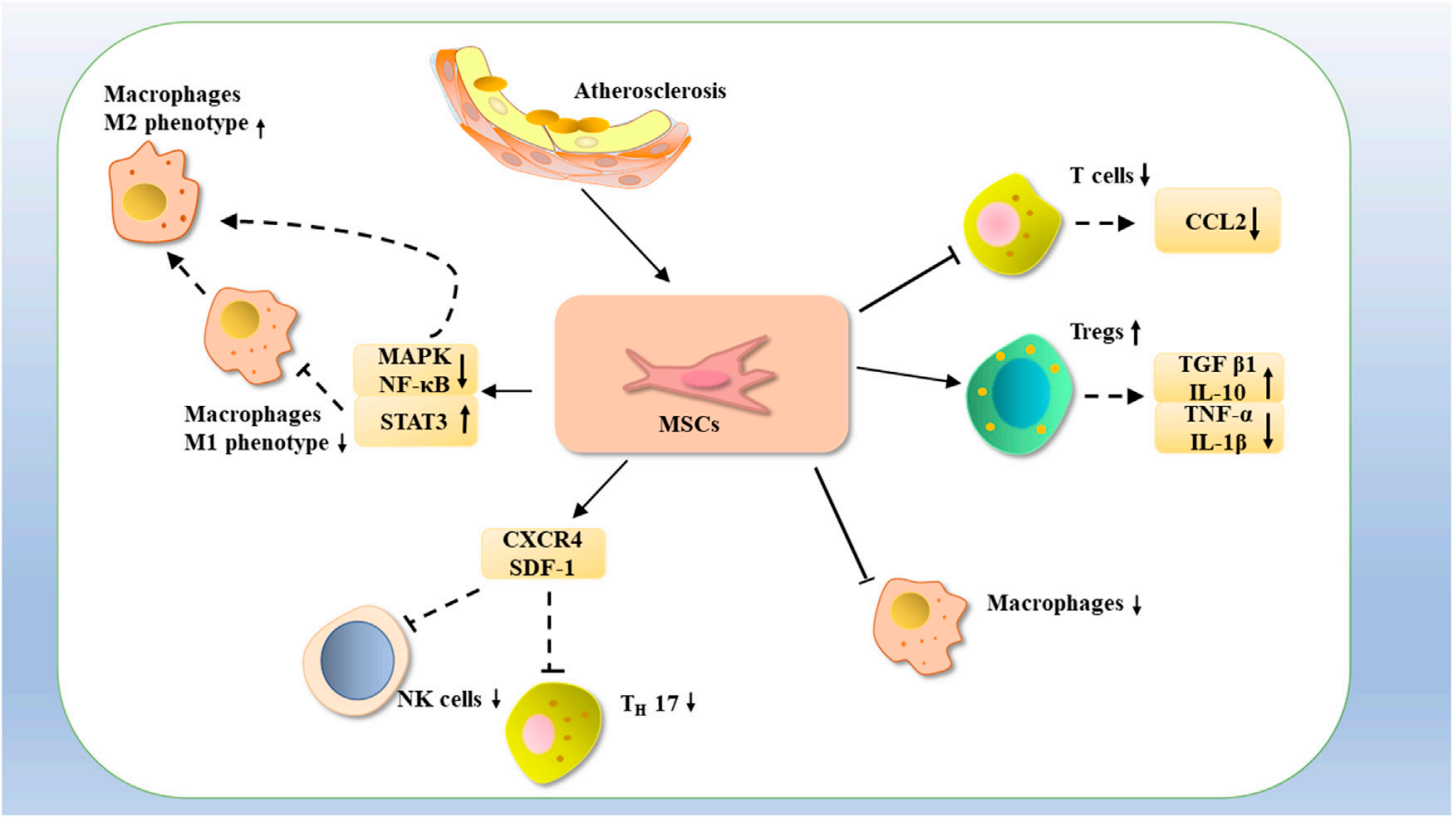
MSCs may treat atherosclerosis by inhibiting the proliferation of T cells with CCL2 expression, or by increasing the number of Tregs, increasing the expression of TGF-β1 and IL-10, decreasing the expression of TNF-α, and IL-1β, as well as by reducing the number of macrophages. Atherosclerosis could be treated by inhibiting NK cells with TH17 through CXCR4 and SDF-1 and downregulating MAPK and NF-κB and upregulating STAT3 expression, thereby inhibiting NK cells M1-type macrophages and promoting the phenotypic transformation of M1-type macrophages to M2-type macrophages.

MSCs are capable of exerting immunomodulatory and immunosuppressive effects through some signaling pathways. Culture media for ADMSCs, like MSCs, can reduce the expression of cell adhesion factors by inhibiting the mitogen-activated protein kinase (MAPK) and NF-κB pathways and reduce the accumulation of macrophages in the vessel wall by inhibiting MAPK and NF-κB pathways. The κB pathway decreases LPS-induced M1 marker expression and increases M2 marker expression through activation of the STAT3 signaling pathway (Takafuji et al., 2019) (Figure 4).

### 4.4 Myocardial Ischemia Reperfusion Injury

After MI, timely reperfusion treatment can reduce the infarct area, but symptoms such as arrhythmia and myocardial contractile dysfunction often occur after reperfusion therapy, thus leading to myocardial ischemia-reperfusion injury (MIRI) (Cohn et al., 2000). Infiltration of inflammatory cells occurs after myocardial ischemia/reperfusion, allowing the progression of MIRI toward inflammatory injury.

Toll-like receptor 2 (TLR2), the most widely expressed member of the TLR family, is the type I transmembrane protein that acts as a pattern recognition receptor, recognizes and binds to several pathogen-associated molecular patterns, and triggers a cascade of signaling that leads to the release of mediators of inflammation, thereby initiating an innate immune response. TLR2 is associated with inflammation and myocardial dysfunction after MIRI, knockdown of MSCs-derived exosomes of TLR2 could improve myocardial recovery from immunomodulation and promote the release of vascular growth factors, thereby protecting cardiac function (Ma et al., 2018). Neutrophils play an important role in the development of MIRI. MSCs were injected in MIRI model rats and were found to be able to initiate an immune response and improve cardiac function in rats after MIRI by enhancing M2 macrophages-induced efferocytosis of apoptotic neutrophils (Zhang et al., 2020a). CD73 can convert Adenosine monophosphate (AMP) into adenosine, thereby inhibiting T-cell activation. In the rat model of MIRI, MSCs were injected into the damaged myocardium and found to mediate the activity of CD73 and attenuate the infiltration of innate immune cells, thereby protecting cardiac function (Shin et al., 2018).

MSCs injection into mice with AMI revealed that MSCs-derived exosomes had profound immunomodulatory effects on DCs and monocytes or macrophages, and exosomes from highly expressed miRNA-181 MSCs created an anti-inflammatory environment, enhanced Tregs polarization, and significantly improved ischemia-reperfusion through downstream c- Fos proteins cardiac function and infarct size in mice with impaired perfusion (Wei et al., 2019). MSCs-derived exosomes could promote the conversion of macrophages from M1 to M2 phenotype by secreting miRNA 182-a and downregulating TLR4, thereby reducing the infarct size and decreasing the level of inflammation in the ischemic myocardium of mice with MIRI (Zhao et al., 2019). The MSCs-derived exosomes were modified with monocyte mimics to form monocyte mimic-bioinspired MSC-EVs (Mon-Exos), which found to have better targeting to the damaged myocardium. In a rat MIRI model, it promoted vascular endothelial repair and modulated macrophage subsets, thus resulted a significant improvement in cardiac function (Zhang et al., 2020b).

Heart transplantation cause MIRI due to procurement and long transplantation times, and although MSCs are cardioprotective, there are still many limitations to this treatment. To address the limitation that MSCs are difficult to transport and store, the researchers treated mice hearts with ischemic cryopreservation followed by the use of a preservation solution, It was found that MSCs-CMs or MSCs-EVs in the preservation solution reversed the adverse effects of long-term frozen ischemia on the donor heart and achieved protection of the donor heart by inhibiting the secretion of pro-inflammatory factors such as IL-1β and IL-6 (Wang et al., 2020). Therefore, both MSCs and MSCs-derived exosomes can inhibit MIRI by exerting their immunoregulatory functions.

### 4.5 Cardiomyopathy and Myocarditis

Dilated cardiomyopathy is defined by dilatation and systolic dysfunction of the left ventricle in the absence of severe coronary artery diseases or abnormal loading conditions. And it is one of the causes of heart failure (Japp et al., 2016). The expression of some genes is extremely important for the immunomodulatory process of MSCs, and MSCs are able to modulate the expression of some cytokines by regulating the expression of genes, thus enabling MSCs to play a role in the treatment of diabetic cardiomyopathy. In the rat model of dilated cardiomyopathy, double overexpression of miR-19a and miR-20a in human-induced iPSC -MSCs was able to suppress the inflammatory response and promote the recovery of cardiac function in rats with dilated cardiomyopathy by inhibiting the secretion of TNF-α/IL-1ß (Sheu et al., 2021). MSCs were injected intravenously into rats with diabetic cardiomyopathy and found to secrete cytokine PGE2 through paracrine action, thereby immunomodulating diabetic cardiomyopathy rats, reducing myocardial fibrosis and alleviating cardiac function (Jin et al., 2020). MSCs-derived exosomes were able to improve the myocardial inflammatory microenvironment in mice with dilated cardiomyopathy by significantly reducing M1 macrophages in the blood and heart and promoting the conversion of macrophages from the M1 phenotype to the M2 phenotype, promoting the recovery of cardiac function (Sun et al., 2018).

Chronic chagas disease cardiomyopathy, a highly fatal inflammatory cardiomyopathy associated with the patient’s innate immune response, mainly characterized by high expression of the pro-inflammatory cytokine Th1 T cells, hypertrophy of cardiomyocytes and prominent fibrosis in the cardiac lesion region (Cunha-Neto and Chevillard. 2014). MSCs may treat Chronic chagas disease cardiomyopathy by regulating the secretion of inflammatory factors. The study showed that MSCs caused inhibition of cardiac inflammation and fibrosis and reduced the expression levels of TNF α, IL-1β, IL-6, and IFN-γ in the mice model of chronic chagas disease cardiomyopathy, and indicated that MSCs had rich potential to treat chronic Chagas disease cardiomyopathy (Souza et al., 2017). MSCs that overexpress granulocyte colony-stimulating factor (MSC G-CSF) could modulate the adaptive immunity of the body by regulating Tregs, leading to the treatment of inflammatory cardiomyopathy caused by chronic Chagas disease (Silva et al., 2018).

**FIGURE 5:**
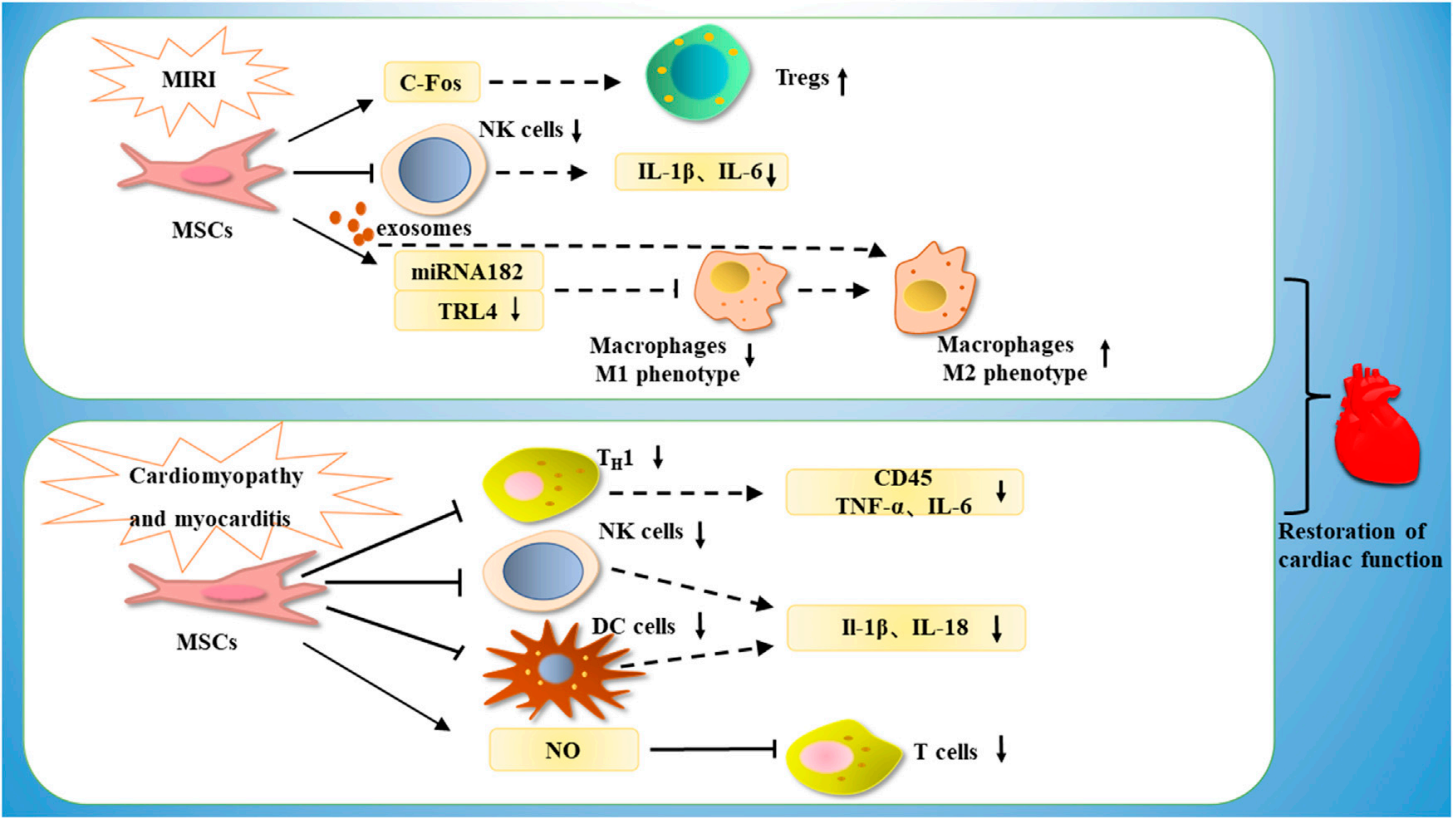
MSCs are able to treat cardiomyopathy by increasing the number of Tregs through c-Fos, or by inhibiting NK cells, decreasing the expression of IL-1β and IL-6, or by releasing miRNA182-a through exosomes, thereby downregulating TRL4, inhibiting M1-type macrophages and promoting phenotypic conversion of M1-type macrophages to M2-type macrophages. In the treatment of myocarditis, MSCs are able to exert immunomodulatory effects by downregulating Th1, thereby downregulating CD45, TNF-α, IL-6, or by inhibiting NK cells and DC cells, thereby downregulating IL-1β and IL-18 expression, or by inhibiting T cell proliferation through NO.

Myocarditis is an inflammatory disease of the myocardium associated with immune dysfunction that causes cardiogenic shock and death (Cooper. 2009). Researchers found that FM-MSCs-treated mice with experimental immune myocarditis had significantly fewer infiltrating Th17 cells and a significantly lower proportion of Th1 cells. It was found that FM-MSCs could improve the body’s immunity by suppressing Th1/Th17, thereby treating experimental immune myocarditis (Ohshima et al., 2012). In addition, MSCs could reduce the severity of experimental immune myocarditis by releasing HGF and inhibiting the expression of IL-2, IL-6 in the myocardium (Okada et al., 2007). Injected MSCs overexpressing IL-10 in a mouse model of autoimmune myocarditis and found that MSCs were able to reduce the inflammatory level of the heart and the degree of myocardial fibrosis by delivering IL-10, thereby preventing and treating overactivated immune system from attacking cardiomyocytes (Shao et al., 2020). Coxsackievirus B3(CVB3) can induce myocarditis. Co-cultured of MSCs with CVB3-infected HL-1 cardiomyocytes revealed that MSCs reduced the CVB3-induced CD4^+^ and CD8^+^ T cells activation in a nitric oxide (NO)-dependent way and required IFN-γ priming (Van Linthout et al., 2011) (Figure 5; Table 2).

**TABLE 2:** Immunomodulatory effect of MSCs in cardiovascular.

Type of Diseases	The Immunomodulatory Modalities of MSCs	Impact	References
Myocardial infarction	Reduction of neutrophils and NK cells expression	Improvement in infarct size, LVEF, and adverse left ventricular remodeling	Luger et al. (2017)
	Inhibition of T-cells proliferation and promotion of macrophages transformation	Enhancement of LVEF and reduction of myocardial fibrosis	(Chen et al., 2019b)
	Promotion of phenotypic conversion of macrophages	Inhibition inflammation-induced injury	(Peng et al., 2016)
	Inhibition of inflammatory response through IL-10	Enhancement of cardiomyocytes viability and reduction of cardiomyocytes apoptosis	(Meng et al., 2018)
	Decrease in macrophages and monocytes levels, promotion of IL-10 expression and decreased IL-1β and IL-6 expression	Improvement of short-term cardiac function and decrease in long-term cardiac remodeling	Dayan et al. (2011)
	Inhibition of lymphocytes proliferation and antibody production *in vitro*	Inhibition of antibody-mediated immune responses to avoid the occurrence of adverse ventricular remodeling	van den Hoogen et al. (2019)
	Increase in Tregs and promotion of IL-10 expression	Improvement of cardiomyocytes microenvironment	(Yoshida et al., 2020)
Heart failure	Reduction of macrophages infiltration and expression of IL-1β and IL-6	Inhibition of myocardial fibrosis and reduction of inflammatory reflection	(Sava et al., 2020)
	Secretion of HGF, inhibition of T cells proliferation, and reduction of Th1, Th2 and cytotoxic T cells production	Improvement of left ventricular function and quality of life in patients	Bartolucci et al. (2017)
	Inhibition of DCs and Th1/Th2 cells function	Improvement of endothelial function and reparation of broken plaques	Li et al. (2017)
	Reduction in the expression of TNF-α and IL-6 and increase in the expression of IL-10		
Atherosclerosis	Inhibition of macrophages by inhibiting MAPK signaling and NF-κB signaling pathways	Reduction of atherosclerotic plaque, decrease in the expression of cells adhesion molecules (CAMs) and the accumulation of macrophages on the vascular walls	Takafuji et al. (2019)
	Regulation of macrophages polarization, inhibition of TNF-α, and increase in IL-10 expression	Inhibition of atheromatous plaque formation	Li et al. (2015b)
	Suppression of T-cells maturation by miRNA-181a	Reduction of ejection fraction, and reduction of infarct size	(Wei et al., 2019)
MIRI	Reduction of IL-6 expression through STAT3	Inhibition of inflammatory response and alleviation of apoptosis in cardiac myocytes	(Poynter et al., 2011)
	Regulation of macrophages polarization and reduction of inflammatory factor levels	Improvement of the inflammatory microenvironment in dilated cardiomyopathy	Sun et al. (2018)
Cardiomyopathy	Reduction of IL-1β, IL-6, and TNF-α expression by Gal-3	Inhibition of inflammation and fibrosis in the heart	(Souza et al., 2017)
	Inhibition of differentiation of Th1 and Th17 cells	Reduction of ventricular ejection fraction	Ohshima et al. (2012)
Myocarditis	Regulation of Th17 phenotype through TGF-β	Improvement of myocardial contractile function, promotion of vascular renewal, and inhibition of cellular inflammatory factors	(Okada et al., 2007)

## 5 Conclusion

MSCs are multipotent stem cells with self-renewal capacity and multidirectional differentiation. They represent the most promising direction for the treatment of diseases such as inflammatory, neurological, cardiovascular, and autoimmune diseases because of their multilineage differentiation potential, immunomodulatory properties, and pro-angiogenic properties (Harrell et al., 2019b). Moreover, its secretory products, such as exosomes, play an essential role in multilineage differentiation function. MSCs have potent immunomodulatory and anti-inflammatory activities, which are mediated by paracrine function and contribute to tissue repair (Zhang et al., 2019). Therefore, MSCs have remarkable efficacy in the treatment of cardiovascular diseases. For example, MSCs mediate cardiomyocytes immune responses through STAT3 to improve cardiac function, regulate c-Fos protein by secreting exosomes and enhance Tregs polarization to improve cardiac function in ischemia-reperfusion injured mice, reduce inflammatory response in the MI area by increasing IL-10 levels, and treat atherosclerosis by regulating the phenotypic transition between macrophage inflammation and phenotype. MSCs have a wide potential for clinical application, which may be related to the alternation of cytokines/cell lineages profiles by lived MSCs surrounding the damaged heart, and both autologous hAM-MSCs and autologous BMMSCs transplantation have also shown good myocardial protection. The immunomodulation of MSCs has a vital role in the repair of myocardial tissue, in addition, the regulation of innate and acquired immunity by MSCs and their derived materials has a place in the treatment of cardiovascular diseases.

In experimental and clinical treatments, MSCs have been studied for immunomodulation mostly by *in vitro* culture and intravenous infusion (currently, there are also clinical cases of nebulized inhalation of MSCs to treat COVID-19, and immunomodulation by MSCs is also a new direction for future research on COVID-19) (Yen et al., 2020; Hong and Hashimoto. 2021). Different MSCs preparation methods also have an impact on the immunomodulatory effects of MSCs. The preparation methods of MSCs include flat plate preparation method, stirred bioreactor preparation method (Eibes et al., 2010), perfusion bioreactor preparation method, etc. (Cho et al., 2008). However, the stirred bioreactor preparation method may lead to cells destruction and thus affect the immunomodulatory activity of MSCs. MSCs exert immune regulation mostly through direct cell-to-cell contact, for example, promoting phenotypic transformation of macrophages through direct cell-to-cell contact (Terai et al., 2021) or by releasing cytokines through paracrine effect, such as secretion, IDO, TGF-β, and PGE2 (Di Trapani et al., 2016). It can function as an immunomodulatory agent by secreting exosomes, such as the inhibition of IL-6 and TNF-α secretion through exosomes, thereby regulating the inflammatory microenvironment (Lu et al., 2021). Nevertheless, MSCs still have many immunomodulatory potentials and require our findings.

According to the current study, there are still some limitations in the clinical application of MSCs. For example, unexpected differentiation, low *in vivo* survival rate of transplanted, and calcification of the infarcted area affect the long-term outcome of MI. The storage and transportation of MSCs is also the reason for the limited clinical application. Studies have shown that the cryopreservation of MSCs can lead to the damage of their immunoregulatory characteristics (Namba. 2019). Therefore, although the immunomodulatory function of MSCs may be a breakthrough and promising therapeutic approach for the treatment of cardiovascular diseases, it is still in a validated state, and further basic research and clinical data are needed to ensure the feasibility of MSCs in the treatment of cardiovascular diseases.

## References

[B1] Adutler-Lieber S. Ben-Mordechai T. Naftali-Shani N. Asher E. Loberman D. Raanani E. (2013). Human Macrophage Regulation via Interaction with Cardiac Adipose Tissue-Derived Mesenchymal Stromal Cells. J. Cardiovasc. Pharmacol. Ther. 18, 78–86. 10.1177/1074248412453875 22894882

[B2] Albany C. J. Trevelin S. C. Giganti G. Lombardi G. Scottà C. (2019). Getting to the Heart of the Matter: The Role of Regulatory T-Cells (Tregs) in Cardiovascular Disease (CVD) and Atherosclerosis. Front. Immunol. 10, 2795. 10.3389/fimmu.2019.02795 31849973 PMC6894511

[B3] Angioni R. Calì B. Vigneswara V. Crescenzi M. Merino A. Sánchez-Rodríguez R. (2020). Administration of Human MSC-Derived Extracellular Vesicles for the Treatment of Primary Sclerosing Cholangitis: Preclinical Data in MDR2 Knockout Mice. Ijms 21, 8874. 10.3390/ijms21228874 PMC7700340 33238629

[B4] Asari S. Itakura S. Ferreri K. Liu C.-P. Kuroda Y. Kandeel F. (2009). Mesenchymal Stem Cells Suppress B-Cell Terminal Differentiation. Exp. Hematol. 37, 604–615. 10.1016/j.exphem.2009.01.005 19375651 PMC2747661

[B5] Bartolucci J. Verdugo F. J. González P. L. Larrea R. E. Abarzua E. Goset C. (2017). Safety and Efficacy of the Intravenous Infusion of Umbilical Cord Mesenchymal Stem Cells in Patients with Heart Failure. Circ. Res. 121, 1192–1204. 10.1161/circresaha.117.310712 28974553 PMC6372053

[B6] Bradley T. Peppa D. Pedroza-Pacheco I. Li D. Cain D. W. Henao R. (2018). RAB11FIP5 Expression and Altered Natural Killer Cell Function Are Associated with Induction of HIV Broadly Neutralizing Antibody Responses. Cell 175, 387–399. 10.1016/j.cell.2018.08.064 30270043 PMC6176872

[B7] Briseño C. G. Murphy T. L. Murphy K. M. (2014). Complementary Diversification of Dendritic Cells and Innate Lymphoid Cells. Curr. Opin. Immunol. 29, 69–78. 10.1016/j.coi.2014.04.006 24874447 PMC5161034

[B8] Butler J. Epstein S. E. Greene S. J. Quyyumi A. A. Sikora S. Kim R. J. (2017). Intravenous Allogeneic Mesenchymal Stem Cells for Nonischemic Cardiomyopathy. Circ. Res. 120, 332–340. 10.1161/circresaha.116.309717 27856497

[B9] Cayrol C. Girard J.-P. (2018). Interleukin-33 (IL-33): A Nuclear Cytokine from the IL-1 Family. Immunol. Rev. 281, 154–168. 10.1111/imr.12619 29247993

[B10] Chachques J. C. Gardin C. Lila N. Ferroni L. Migonney V. Falentin-Daudre C. (2021). Elastomeric Cardiowrap Scaffolds Functionalized with Mesenchymal Stem Cells-Derived Exosomes Induce a Positive Modulation in the Inflammatory and Wound Healing Response of Mesenchymal Stem Cell and Macrophage. Biomedicines 9, 824. 10.3390/biomedicines9070824 34356888 PMC8301323

[B11] Chen X. Cai C. Xu D. Liu Q. Zheng S. Liu L. (2019a). Human Mesenchymal Stem Cell-Treated Regulatory CD23+CD43+ B Cells Alleviate Intestinal Inflammation. Theranostics 9, 4633–4647. 10.7150/thno.32260 31367246 PMC6643430

[B12] Chen Y. Zuo J. Chen W. Yang Z. Zhang Y. Hua F. (2019b). The Enhanced Effect and Underlying Mechanisms of Mesenchymal Stem Cells with IL-33 Overexpression on Myocardial Infarction. Stem Cel Res Ther 10, 295. 10.1186/s13287-019-1392-9 PMC6757387 31547872

[B13] Cho C. H. Eliason J. F. Matthew H. W. T. (2008). Application of Porous Glycosaminoglycan-Based Scaffolds for Expansion of Human Cord Blood Stem Cells in Perfusion Culture. J. Biomed. Mater. Res. 86A, 98–107. 10.1002/jbm.a.31614 17941019

[B14] Cho D.-I. Kim M. R. Jeong H.-y. Jeong H. C. Jeong M. H. Yoon S. H. (2014). Mesenchymal Stem Cells Reciprocally Regulate the M1/M2 Balance in Mouse Bone Marrow-Derived Macrophages. Exp. Mol. Med. 46, e70. 10.1038/emm.2013.135 24406319 PMC3909888

[B15] Clark G. J. Silveira P. A. Hogarth P. M. Hart D. N. J. (2019). The Cell Surface Phenotype of Human Dendritic Cells. Semin. Cel Develop. Biol. 86, 3–14. 10.1016/j.semcdb.2018.02.013 29499385

[B16] Cohn J. N. Ferrari R. Sharpe N. (2000). Cardiac Remodeling-Concepts and Clinical Implications: a Consensus Paper from an International Forum on Cardiac Remodeling. J. Am. Coll. Cardiol. 35, 569–582. 10.1016/s0735-1097(99)00630-0 10716457

[B17] Consentius C. Akyüz L. Schmidt‐Lucke J. A. Tschöpe C. Pinzur L. Ofir R. (2015). Mesenchymal Stromal Cells Prevent Allostimulation *In Vivo* and Control Checkpoints of Th1 Priming: Migration of Human DC to Lymph Nodes and NK Cell Activation. Stem Cells 33, 3087–3099. 10.1002/stem.2104 26184374

[B18] Cooper L. T. Jr. (2009). Myocarditis. N. Engl. J. Med. 360, 1526–1538. 10.1056/NEJMra0800028 19357408 PMC5814110

[B19] Cui R. Rekasi H. Hepner-Schefczyk M. Fessmann K. Petri R. M. Bruderek K. (2016). Human Mesenchymal Stromal/stem Cells Acquire Immunostimulatory Capacity upon Cross-Talk with Natural Killer Cells and Might Improve the NK Cell Function of Immunocompromised Patients. Stem Cel Res Ther 7, 88. 10.1186/s13287-016-0353-9 PMC4937587 27388156

[B20] Cunha-Neto E. Chevillard C. (2014). Chagas Disease Cardiomyopathy: Immunopathology and Genetics. Mediators Inflamm. 2014, 1–11. 10.1155/2014/683230 PMC4152981 25210230

[B21] Daneshmandi S. Karimi M. H. Pourfathollah A. A. (2017). TGF-β1 Transduced Mesenchymal Stem Cells Have Profound Modulatory Effects on DCs and T Cells. Iran J. Immunol. 14, 13–23. 28341815 10.22034/iji.2017.39285

[B22] Dayan V. Yannarelli G. Billia F. Filomeno P. Wang X.-H. Davies J. E. (2011). Mesenchymal Stromal Cells Mediate a Switch to Alternatively Activated Monocytes/macrophages after Acute Myocardial Infarction. Basic Res. Cardiol. 106, 1299–1310. 10.1007/s00395-011-0221-9 21901289

[B23] Di Trapani M. Bassi G. Midolo M. Gatti A. Takam Kamga P. Cassaro A. (2016). Differential and Transferable Modulatory Effects of Mesenchymal Stromal Cell-Derived Extracellular Vesicles on T, B and NK Cell Functions. Sci. Rep. 6, 24120. 10.1038/srep24120 27071676 PMC4829861

[B24] Ding D.-C. Shyu W.-C. Lin S.-Z. (2011). Mesenchymal Stem Cells. Cel Transpl. 20, 5–14. 10.3727/096368910x 21396235

[B25] Dong B. Wang C. Zhang J. Zhang J. Gu Y. Guo X. (2021). Exosomes from Human Umbilical Cord Mesenchymal Stem Cells Attenuate the Inflammation of Severe Steroid-Resistant Asthma by Reshaping Macrophage Polarization. Stem Cel Res Ther 12, 204. 10.1186/s13287-021-02244-6 PMC7988945 33761997

[B26] Eibes G. dos Santos F. Andrade P. Z. Boura J. S. Abecasis M. M. A. da Silva C. L. (2010). Maximizing the *Ex Vivo* Expansion of Human Mesenchymal Stem Cells Using a Microcarrier-Based Stirred Culture System. J. Biotechnol. 146, 194–197. 10.1016/j.jbiotec.2010.02.015 20188771

[B27] Feng Y. Xu Q. Yang Y. Shi W. Meng W. Zhang H. (2019). The Therapeutic Effects of Bone Marrow-Derived Mesenchymal Stromal Cells in the Acute Lung Injury Induced by Sulfur Mustard. Stem Cel Res Ther 10, 90. 10.1186/s13287-019-1189-x PMC6416968 30867053

[B28] Fields L. Ito T. Kobayashi K. Ichihara Y. Podaru M.-N. Hussain M. (2021). Epicardial Placement of Human MSC-Loaded Fibrin Sealant Films for Heart Failure: Preclinical Efficacy and Mechanistic Data. Mol. Ther. 29, 2554–2570. 10.1016/j.ymthe.2021.04.018 33887461 PMC8353205

[B29] Ghosh T. Barik S. Bhuniya A. Dhar J. Dasgupta S. Ghosh S. (2016). Tumor-associated Mesenchymal Stem Cells Inhibit Naïve T Cell Expansion by Blocking Cysteine export from Dendritic Cells. Int. J. Cancer 139, 2068–2081. 10.1002/ijc.30265 27405489

[B30] Giri J. Das R. Nylen E. Chinnadurai R. Galipeau J. (2020). CCL2 and CXCL12 Derived from Mesenchymal Stromal Cells Cooperatively Polarize IL-10+ Tissue Macrophages to Mitigate Gut Injury. Cel Rep. 30, 1923–1934. 10.1016/j.celrep.2020.01.047 PMC7043065 32049021

[B31] Gomzikova M. Kletukhina S. Kurbangaleeva S. Neustroeva O. Vasileva O. Garanina E. (2020). Mesenchymal Stem Cell Derived Biocompatible Membrane Vesicles Demonstrate Immunomodulatory Activity Inhibiting Activation and Proliferation of Human Mononuclear Cells. Pharmaceutics 12, 577. 10.3390/pharmaceutics12060577 PMC7356506 32585863

[B32] Goodwin M. Sueblinvong V. Eisenhauer P. Ziats N. P. LeClair L. Poynter M. E. (2011). Bone Marrow‐Derived Mesenchymal Stromal Cells Inhibit Th2‐Mediated Allergic Airways Inflammation in Mice. Stem Cells 29, 1137–1148. 10.1002/stem.656 21544902 PMC4201366

[B33] Gupte K. S. Vanikar A. V. Trivedi H. L. Patel C. N. Patel J. V. (2017). *In-vitro* Generation of Interleukin-10 Secreting B-Regulatory Cells from Donor Adipose Tissue Derived Mesenchymal Stem Cells and Recipient Peripheral Blood Mononuclear Cells for Potential Cell Therapy. Biomed. J. 40, 49–54. 10.1016/j.bj.2017.01.003 28411882 PMC6138595

[B34] Hansson G. K. Hermansson A. (2011). The Immune System in Atherosclerosis. Nat. Immunol. 12, 204–212. 10.1038/ni.2001 21321594

[B35] Harrell C. Fellabaum C. Jovicic N. Djonov V. Arsenijevic N. Volarevic V. (2019b). Molecular Mechanisms Responsible for Therapeutic Potential of Mesenchymal Stem Cell-Derived Secretome. Cells 8, 467. 10.3390/cells8050467 PMC6562906 31100966

[B36] Harrell C. R. Jovicic N. Djonov V. Arsenijevic N. Volarevic V. (2019a). Mesenchymal Stem Cell-Derived Exosomes and Other Extracellular Vesicles as New Remedies in the Therapy of Inflammatory Diseases. Cells 8, 1605. 10.3390/cells8121605 PMC6952783 31835680

[B37] He H. Liu X. Peng L. Gao Z. Ye Y. Su Y. (2013). Promotion of Hepatic Differentiation of Bone Marrow Mesenchymal Stem Cells on Decellularized Cell-Deposited Extracellular Matrix. Biomed. Res. Int. 2013, 1–11. 10.1155/2013/406871 PMC3749543 23991414

[B38] Hodgins J. J. Khan S. T. Park M. M. Auer R. C. Ardolino M. (2019). Killers 2.0: NK Cell Therapies at the Forefront of Cancer Control. J. Clin. Invest. 129, 3499–3510. 10.1172/jci129338 31478911 PMC6715409

[B39] Hong Y. Hashimoto M. (2021). I Will Get Myself Vaccinated for Others: The Interplay of Message Frame, Reference Point, and Perceived Risk on Intention for COVID-19 Vaccine. Health Commun., 1–11. 10.1080/10410236.2021.1978668 34544315

[B40] Huang P. Wang L. Li Q. Xu J. Xu J. Xiong Y. (2019). Combinatorial Treatment of Acute Myocardial Infarction Using Stem Cells and Their Derived Exosomes Resulted in Improved Heart Performance. Stem Cel Res Ther 10, 300. 10.1186/s13287-019-1353-3 PMC6785902 31601262

[B41] Ichihara Y. Kaneko M. Yamahara K. Koulouroudias M. Sato N. Uppal R. (2018). Self-assembling Peptide Hydrogel Enables Instant Epicardial Coating of the Heart with Mesenchymal Stromal Cells for the Treatment of Heart Failure. Biomaterials 154, 12–23. 10.1016/j.biomaterials.2017.10.050 29117575 PMC5768325

[B42] Ishida N. Ishiyama K. Saeki Y. Tanaka Y. Ohdan H. (2019). Cotransplantation of Preactivated Mesenchymal Stem Cells Improves Intraportal Engraftment of Islets by Inhibiting Liver Natural Killer Cells in Mice. Am. J. Transpl. 19, 2732–2745. 10.1111/ajt.15347 30859713

[B43] J M. P. Debabrata B. (2017). Activation and Differentiation of Mesenchymal Stem Cells. J Methods Mol. Biol. (Clifton, N.J.) 1554. 10.1007/978-1-4939-6759-9_13 28185193

[B44] Japp A. G. Gulati A. Cook S. A. Cowie M. R. Prasad S. K. (2016). The Diagnosis and Evaluation of Dilated Cardiomyopathy. J. Am. Coll. Cardiol. 67, 2996–3010. 10.1016/j.jacc.2016.03.590 27339497

[B45] Jin L. Deng Z. Zhang J. Yang C. Liu J. Han W. (2019). Mesenchymal Stem Cells Promote Type 2 Macrophage Polarization to Ameliorate the Myocardial Injury Caused by Diabetic Cardiomyopathy. J. Transl Med. 17, 251. 10.1186/s12967-019-1999-8 31382970 PMC6683374

[B46] Jin L. Zhang J. Deng Z. Liu J. Han W. Chen G. (2020). Mesenchymal Stem Cells Ameliorate Myocardial Fibrosis in Diabetic Cardiomyopathy via the Secretion of Prostaglandin E2. Stem Cel Res Ther 11, 122. 10.1186/s13287-020-01633-7 PMC7079514 32183879

[B47] Jo H. Eom Y. W. Kim H.-S. Park H. J. Kim H. M. Cho M.-Y. (2018). Regulatory Dendritic Cells Induced by Mesenchymal Stem Cells Ameliorate Dextran Sodium Sulfate-Induced Chronic Colitis in Mice. Gut and Liver 12, 664–673. 10.5009/gnl18072 29938461 PMC6254613

[B48] Kanelidis A. J. Premer C. Lopez J. Balkan W. Hare J. M. (2017). Route of Delivery Modulates the Efficacy of Mesenchymal Stem Cell Therapy for Myocardial Infarction. Circ. Res. 120, 1139–1150. 10.1161/circresaha.116.309819 28031416 PMC5656247

[B49] Koelwyn G. J. Corr E. M. Erbay E. Moore K. J. (2018). Regulation of Macrophage Immunometabolism in Atherosclerosis. Nat. Immunol. 19, 526–537. 10.1038/s41590-018-0113-3 29777212 PMC6314674

[B50] Kumar B. V. Connors T. J. Farber D. L. (2018). Human T Cell Development, Localization, and Function throughout Life. Immunity 48, 202–213. 10.1016/j.immuni.2018.01.007 29466753 PMC5826622

[B51] Lee W. Wang L. T. Yen M. L. Hsu P. J. Lee Y. W. Liu K. J. (2021). Resident vs Nonresident Multipotent Mesenchymal Stromal Cell Interactions with B Lymphocytes Result in Disparate Outcomes. Stem Cell Transl Med 10, 711–724. 10.1002/sctm.20-0289 PMC8046079 33506633

[B52] Lee Y. El Andaloussi S. Wood M. J. A. (2012). Exosomes and Microvesicles: Extracellular Vesicles for Genetic Information Transfer and Gene Therapy. Hum. Mol. Genet. 21, R125–R134. 10.1093/hmg/dds317 22872698

[B53] Lee Y. S. Radford K. J. (2019). The Role of Dendritic Cells in Cancer. Int. Rev. Cel Mol Biol 348, 123–178. 10.1016/bs.ircmb.2019.07.006 31810552

[B54] Li C. Jin Y. Wei S. Sun Y. Jiang L. Zhu Q. (2019). Hippo Signaling Controls NLR Family Pyrin Domain Containing 3 Activation and Governs Immunoregulation of Mesenchymal Stem Cells in Mouse Liver Injury. Hepatology 70, 1714–1731. 10.1002/hep.30700 31063235 PMC6819196

[B55] Li F. Guo X. Chen S.-Y. (2017). Function and Therapeutic Potential of Mesenchymal Stem Cells in Atherosclerosis. Front. Cardiovasc. Med. 4, 32. 10.3389/fcvm.2017.00032 28589127 PMC5438961

[B56] Li Q. Sun W. Wang X. Zhang K. Xi W. Gao P. (2015b). Skin-Derived Mesenchymal Stem Cells Alleviate Atherosclerosis via Modulating Macrophage Function. Stem Cell Transl Med 4, 1294–1301. 10.5966/sctm.2015-0020 PMC4622403 26400926

[B57] Li T. Xia M. Gao Y. Chen Y. Xu Y. (2015a). Human Umbilical Cord Mesenchymal Stem Cells: an Overview of Their Potential in Cell-Based Therapy. Expert Opin. Biol. Ther. 15, 1293–1306. 10.1517/14712598.2015.1051528 26067213

[B58] Liao S. Zhang Y. Ting S. Zhen Z. Luo F. Zhu Z. (2019). Potent Immunomodulation and Angiogenic Effects of Mesenchymal Stem Cells versus Cardiomyocytes Derived from Pluripotent Stem Cells for Treatment of Heart Failure. Stem Cel Res Ther 10, 78. 10.1186/s13287-019-1183-3 PMC6407247 30845990

[B59] Liénart S. Merceron R. Vanderaa C. Lambert F. Colau D. Stockis J. (2018). Structural Basis of Latent TGF-Β1 Presentation and Activation by GARP on Human Regulatory T Cells. Science 362, 952–956. 10.1126/science.aau2909 30361387

[B60] Liu F. Qiu H. Xue M. Zhang S. Zhang X. Xu J. (2019). MSC-secreted TGF-β Regulates Lipopolysaccharide-Stimulated Macrophage M2-like Polarization via the Akt/FoxO1 Pathway. Stem Cel Res Ther 10, 345. 10.1186/s13287-019-1447-y PMC6878630 31771622

[B61] Liu Z. Mikrani R. Zubair H. M. Taleb A. Naveed M. Baig M. M. F. A. (2020). Systemic and Local Delivery of Mesenchymal Stem Cells for Heart Renovation: Challenges and Innovations. Eur. J. Pharmacol. 876, 173049. 10.1016/j.ejphar.2020.173049 32142771

[B62] Loo T. T. Gao Y. Lazarevic V. (2018). Transcriptional Regulation of CD4+TH Cells that Mediate Tissue Inflammation. J. Leukoc. Biol. 104, 1069–1085. 10.1002/jlb.1ri0418-152rr 30145844 PMC6662913

[B63] Lopatina T. Kalinina N. Karagyaur M. Stambolsky D. Rubina K. Revischin A. (2019). Correction: Adipose-Derived Stem Cells Stimulate Regeneration of Peripheral Nerves: BDNF Secreted by These Cells Promotes Nerve Healing and Axon Growth De Novo. PLoS One 14, e0219946. 10.1371/journal.pone.0219946 31299059 PMC6625725

[B64] Lu H. Zhang Y. Xiong S. Zhou Y. Xiao L. Ma Y. (2021). Modulatory Role of Silver Nanoparticles and Mesenchymal Stem Cell-Derived Exosome-Modified Barrier Membrane on Macrophages and Osteogenesis. Front. Chem. 9, 699802. 10.3389/fchem.2021.699802 34409016 PMC8365089

[B65] Luger D. Lipinski M. J. Westman P. C. Glover D. K. Dimastromatteo J. Frias J. C. (2017). Intravenously Delivered Mesenchymal Stem Cells. Circ. Res. 120, 1598–1613. 10.1161/circresaha.117.310599 28232595

[B66] Luo L. Tang J. Nishi K. Yan C. Dinh P.-U. Cores J. (2017). Fabrication of Synthetic Mesenchymal Stem Cells for the Treatment of Acute Myocardial Infarction in Mice. Circ. Res. 120, 1768–1775. 10.1161/circresaha.116.310374 28298296 PMC5488324

[B67] Lv K. Li Q. Zhang L. Wang Y. Zhong Z. Zhao J. (2019). Incorporation of Small Extracellular Vesicles in Sodium Alginate Hydrogel as a Novel Therapeutic Strategy for Myocardial Infarction. Theranostics 9, 7403–7416. 10.7150/thno.32637 31695776 PMC6831299

[B68] Ma J. Chen L. Zhu X. Li Q. Hu L. Li H. (2021). Mesenchymal Stem Cell-Derived Exosomal miR-21a-5p Promotes M2 Macrophage Polarization and Reduces Macrophage Infiltration to Attenuate Atherosclerosis. Acta Biochim. Biophys. Sin (Shanghai) 53, 1227–1236. 10.1093/abbs/gmab102 34350954

[B69] Ma S. Q. Wei H. L. Zhang X. (2018). TLR2 Regulates Allergic Airway Inflammation through NF-Κb and MAPK Signaling Pathways in Asthmatic Mice. Eur. Rev. Med. Pharmacol. Sci. 22, 3138–3146. 10.26355/eurrev_201805_15073 29863259

[B70] Magatti M. Masserdotti A. Bonassi Signoroni P. Vertua E. Stefani F. R. Silini A. R. (2020). B Lymphocytes as Targets of the Immunomodulatory Properties of Human Amniotic Mesenchymal Stromal Cells. Front. Immunol. 11, 1156. 10.3389/fimmu.2020.01156 32582218 PMC7295987

[B71] Martinez V. G. Ontoria-Oviedo I. Ricardo C. P. Harding S. E. Sacedon R. Varas A. (2017). Overexpression of Hypoxia-Inducible Factor 1 Alpha Improves Immunomodulation by Dental Mesenchymal Stem Cells. Stem Cel Res Ther 8, 208. 10.1186/s13287-017-0659-2 PMC5622468 28962641

[B72] Mauri C. Bosma A. (2012). Immune Regulatory Function of B Cells. Annu. Rev. Immunol. 30, 221–241. 10.1146/annurev-immunol-020711-074934 22224776

[B73] Mekhloufi A. Kosta A. Stabile H. Molfetta R. Zingoni A. Soriani A. (2020). Bone Marrow Stromal Cell-Derived IL-8 Upregulates PVR Expression on Multiple Myeloma Cells via NF-kB Transcription Factor. Cancers 12, 440. 10.3390/cancers12020440 PMC7072437 32069911

[B74] Meng X. Li J. Yu M. Yang J. Zheng M. Zhang J. (2018). Transplantation of Mesenchymal Stem Cells Overexpressing IL10 Attenuates Cardiac Impairments in Rats with Myocardial Infarction. J. Cel Physiol 233, 587–595. 10.1002/jcp.25919 28322445

[B75] Monguió-Tortajada M. Prat-Vidal C. Moron-Font M. Clos-Sansalvador M. Calle A. Gastelurrutia P. (2021). Local Administration of Porcine Immunomodulatory, Chemotactic and Angiogenic Extracellular Vesicles Using Engineered Cardiac Scaffolds for Myocardial Infarction. Bioactive Mater. 6, 3314–3327. 10.1016/j.bioactmat.2021.02.026 PMC7973387 33778207

[B76] Morvan M. G. Lanier L. L. (2016). NK Cells and Cancer: You Can Teach Innate Cells New Tricks. Nat. Rev. Cancer 16, 7–19. 10.1038/nrc.2015.5 26694935

[B77] Namba F. (2019). Mesenchymal Stem Cells for the Prevention of Bronchopulmonary Dysplasia. Pediatr. Int. 61, 945–950. 10.1111/ped.14001 31487104

[B78] Ohshima M. Yamahara K. Ishikane S. Harada K. Tsuda H. Otani K. (2012). Systemic Transplantation of Allogenic Fetal Membrane-Derived Mesenchymal Stem Cells Suppresses Th1 and Th17 T Cell Responses in Experimental Autoimmune Myocarditis. J. Mol. Cell Cardiol. 53, 420–428. 10.1016/j.yjmcc.2012.06.020 22796574

[B79] Okada H. Suzuki J.-i. Futamatsu H. Maejima Y. Hirao K. Isobe M. (2007). Attenuation of Autoimmune Myocarditis in Rats by Mesenchymal Stem Cell Transplantation through Enhanced Expression of Hepatocyte Growth Factor. Int. Heart J. 48, 649–661. 10.1536/ihj.48.649 17998774

[B80] Overgaard N. H. Jung J.-W. Steptoe R. J. Wells J. W. (2015). CD4+/CD8+double-positive T Cells: More Than Just a Developmental Stage? J. Leukoc. Biol. 97, 31–38. 10.1189/jlb.1RU0814-382 25360000

[B81] Özgül Özdemir R. B. Özdemir A. T. Kırmaz C. Eker Sarıboyacı A. Karaöz E. Erman G. (2021). Age-related Changes in the Immunomodulatory Effects of Human Dental Pulp Derived Mesenchymal Stem Cells on the CD4+ T Cell Subsets. Cytokine 138, 155367. 10.1016/j.cyto.2020.155367 33223447

[B82] P. De Miguel M. Fuentes-Julian S. Blazquez-Martinez A. Y. Pascual C. A. Aller M. Arias J. (2012). Immunosuppressive Properties of Mesenchymal Stem Cells: Advances and Applications. Cmm 12, 574–591. 10.2174/156652412800619950 22515979

[B83] Peng Y. Chen X. Liu Q. Xu D. Zheng H. Liu L. (2014). Alteration of Naïve and Memory B-Cell Subset in Chronic Graft-Versus-Host Disease Patients after Treatment with Mesenchymal Stromal Cells. Stem Cell Transl Med 3, 1023–1031. 10.5966/sctm.2014-0001 PMC4149298 25015640

[B84] Peng Y. Pan W. Ou Y. Xu W. Kaelber S. Borlongan C. V. (2016). Extracardiac-Lodged Mesenchymal Stromal Cells Propel an Inflammatory Response against Myocardial Infarction via Paracrine Effects. Cel Transpl. 25, 929–935. 10.3727/096368915x689758 26498018

[B85] Petri R. M. Hackel A. Hahnel K. Dumitru C. A. Bruderek K. Flohe S. B. (2017). Activated Tissue-Resident Mesenchymal Stromal Cells Regulate Natural Killer Cell Immune and Tissue-Regenerative Function. Stem Cel Rep. 9, 985–998. 10.1016/j.stemcr.2017.06.020 PMC5599186 28781075

[B86] Philipp D. Holthaus M. Basoah V. Pfannkuche K. Suhr L. Wahlers T. (2021). VEGF Contributes to Mesenchymal Stem Cell-Mediated Reversion of Nor1-dependent Hypertrophy in iPS Cell-Derived Cardiomyocytes. Stem Cell Int. 2021, 1–19. 10.1155/2021/8888575 PMC8053052 33927770

[B87] Pontikoglou C. Kastrinaki M.-C. Klaus M. Kalpadakis C. Katonis P. Alpantaki K. (2013). Study of the Quantitative, Functional, Cytogenetic, and Immunoregulatory Properties of Bone Marrow Mesenchymal Stem Cells in Patients with B-Cell Chronic Lymphocytic Leukemia. Stem Cell Develop. 22, 1329–1341. 10.1089/scd.2012.0255 PMC3629855 23249221

[B88] Poynter J. A. Herrmann J. L. Manukyan M. C. Wang Y. Abarbanell A. M. Weil B. R. (2011). Intracoronary Mesenchymal Stem Cells Promote Postischemic Myocardial Functional Recovery, Decrease Inflammation, and Reduce Apoptosis via a Signal Transducer and Activator of Transcription 3 Mechanism. J. Am. Coll. Surgeons 213, 253–260. 10.1016/j.jamcollsurg.2011.04.005 21546276

[B89] Qin Y. Zhou Z. Zhang F. Wang Y. Shen B. Liu Y. (2015). Induction of Regulatory B-Cells by Mesenchymal Stem Cells Is Affected by SDF-1α-CXCR7. Cell Physiol Biochem 37, 117–130. 10.1159/000430338 26303308

[B90] Qingqing M. Xin Z. Meizhong S. (2014). Bone Marrow Mesenchymal Stem Cells Altered the Immunoregulatory Activities of Hepatic Natural Killer Cells. Clin. Res. Hepatol. Gastroenterol. 38, 689–698. 10.1016/j.clinre.2014.06.001 25241998

[B91] Rasmusson I. Le Blanc K. Sundberg B. Ringdén O. (2007). Mesenchymal Stem Cells Stimulate Antibody Secretion in Human B Cells. Scand. J. Immunol. 65, 336–343. 10.1111/j.1365-3083.2007.01905.x 17386024

[B92] Riazifar M. Mohammadi M. R. Pone E. J. Yeri A. Lässer C. Segaliny A. I. (2019). Stem Cell-Derived Exosomes as Nanotherapeutics for Autoimmune and Neurodegenerative Disorders. ACS Nano 13, 6670–6688. 10.1021/acsnano.9b01004 31117376 PMC6880946

[B93] Saraiva M. O'Garra A. (2010). The Regulation of IL-10 Production by Immune Cells. Nat. Rev. Immunol. 10, 170–181. 10.1038/nri2711 20154735

[B94] Sava R. Pepine C. March K. (2020). Immune Dysregulation in HFpEF: A Target for Mesenchymal Stem/Stromal Cell Therapy. Jcm 9, 241. 10.3390/jcm9010241 PMC7019215 31963368

[B95] Shao M. Wang D. Zhou Y. Du K. Liu W. (2020). Interleukin-10 Delivered by Mesenchymal Stem Cells Attenuates Experimental Autoimmune Myocarditis. Int. Immunopharmacology 81, 106212. 10.1016/j.intimp.2020.106212 32062070

[B96] Sharma V. Dash S. K. Manhas A. Radhakrishnan J. Jagavelu K. Verma R. S. (2021). Injectable Hydrogel for Co-delivery of 5-azacytidine in Zein Protein Nanoparticles with Stem Cells for Cardiac Function Restoration. Int. J. Pharmaceutics 603, 120673. 10.1016/j.ijpharm.2021.120673 33964338

[B97] Sheu J.-J. Chai H.-T. Sung P.-H. Chiang J. Y. Huang T.-H. Shao P.-L. (2021). Double Overexpression of miR-19a and miR-20a in Induced Pluripotent Stem Cell-Derived Mesenchymal Stem Cells Effectively Preserves the Left Ventricular Function in Dilated Cardiomyopathic Rat. Stem Cel Res Ther 12, 371. 10.1186/s13287-021-02440-4 PMC8243466 34187571

[B98] Shi H. Liang M. Chen W. Sun X. Wang X. Li C. (2018b). Human Induced Pluripotent Stem Cell-derived M-esenchymal S-tem C-ells A-lleviate A-therosclerosis by M-odulating I-nflammatory R-esponses. Mol. Med. Rep. 17, 1461–1468. 10.3892/mmr.2017.8075 29257199 PMC5780084

[B99] Shi Y. Wang Y. Li Q. Liu K. Hou J. Shao C. (2018a). Immunoregulatory Mechanisms of Mesenchymal Stem and Stromal Cells in Inflammatory Diseases. Nat. Rev. Nephrol. 14, 493–507. 10.1038/s41581-018-0023-5 29895977

[B100] Shin E. Y. Wang L. Zemskova M. Deppen J. Xu K. Strobel F. (2018). Adenosine Production by Biomaterial‐Supported Mesenchymal Stromal Cells Reduces the Innate Inflammatory Response in Myocardial Ischemia/Reperfusion Injury. Jaha 7. 10.1161/jaha.117.006949 PMC5850147 29331956

[B101] Shou P. Chen Q. Jiang J. Xu C. Zhang J. Zheng C. (2016). Type I Interferons Exert Anti-tumor Effect via Reversing Immunosuppression Mediated by Mesenchymal Stromal Cells. Oncogene 35, 5953–5962. 10.1038/onc.2016.128 27109100 PMC5079855

[B102] Silva D. N. Souza B. S. F. Vasconcelos J. F. Azevedo C. M. Valim C. X. R. Paredes B. D. (2018). Granulocyte-Colony Stimulating Factor-Overexpressing Mesenchymal Stem Cells Exhibit Enhanced Immunomodulatory Actions through the Recruitment of Suppressor Cells in Experimental Chagas Disease Cardiomyopathy. Front. Immunol. 9, 1449. 10.3389/fimmu.2018.01449 30013550 PMC6036245

[B103] Souza B. S. d. F. Silva K. N. d. Silva D. N. Rocha V. P. C. Paredes B. D. Azevedo C. M. (2017). Galectin-3 Knockdown Impairs Survival, Migration, and Immunomodulatory Actions of Mesenchymal Stromal Cells in a Mouse Model of Chagas Disease Cardiomyopathy. Stem Cell Int. 2017, 1–13. 10.1155/2017/3282656 PMC5523546 28769980

[B104] Spaggiari G. M. Abdelrazik H. Becchetti F. Moretta L. (2009). MSCs Inhibit Monocyte-Derived DC Maturation and Function by Selectively Interfering with the Generation of Immature DCs: central Role of MSC-Derived Prostaglandin E2. Blood 113, 6576–6583. 10.1182/blood-2009-02-203943 19398717

[B105] Spaggiari G. M. Capobianco A. Abdelrazik H. Becchetti F. Mingari M. C. Moretta L. (2008). Mesenchymal Stem Cells Inhibit Natural Killer-Cell Proliferation, Cytotoxicity, and Cytokine Production: Role of Indoleamine 2,3-dioxygenase and Prostaglandin E2. Blood 111, 1327–1333. 10.1182/blood-2007-02-074997 17951526

[B106] Spaggiari G. M. Capobianco A. Becchetti S. Mingari M. C. Moretta L. (2006). Mesenchymal Stem Cell-Natural Killer Cell Interactions: Evidence that Activated NK Cells Are Capable of Killing MSCs, whereas MSCs Can Inhibit IL-2-induced NK-Cell Proliferation. Blood 107, 1484–1490. 10.1182/blood-2005-07-2775 16239427

[B107] Spees J. L. Lee R. H. Gregory C. A. (2016). Mechanisms of Mesenchymal Stem/stromal Cell Function. Stem Cel Res Ther 7, 125. 10.1186/s13287-016-0363-7 PMC5007684 27581859

[B108] Sun X. Shan A. Wei Z. Xu B. (2018). Intravenous Mesenchymal Stem Cell-Derived Exosomes Ameliorate Myocardial Inflammation in the Dilated Cardiomyopathy. Biochem. Biophysical Res. Commun. 503, 2611–2618. 10.1016/j.bbrc.2018.08.012 30126637

[B109] Takafuji Y. Hori M. Mizuno T. Harada-Shiba M. (2019). Humoral Factors Secreted from Adipose Tissue-Derived Mesenchymal Stem Cells Ameliorate Atherosclerosis in Ldlr−/− Mice. Cardiovasc. Res. 115, 1041–1051. 10.1093/cvr/cvy271 30388208

[B110] Tanaka T. Narazaki M. Kishimoto T. (2014). IL-6 in Inflammation, Immunity, and Disease. Cold Spring Harbor Perspect. Biol. 6, a016295. 10.1101/cshperspect.a016295 PMC4176007 25190079

[B111] Terai S. Tsuchiya A. Watanabe Y. Takeuchi S. (2021). Transition of Clinical and Basic Studies on Liver Cirrhosis Treatment Using Cells to Seek the Best Treatment. Inflamm. Regener 41, 27. 10.1186/s41232-021-00178-3 PMC8444392 34530931

[B112] Uccelli A. Moretta L. Pistoia V. (2008). Mesenchymal Stem Cells in Health and Disease. Nat. Rev. Immunol. 8, 726–736. 10.1038/nri2395 19172693

[B113] van den Hoogen P. de Jager S. C. A. Mol E. A. Schoneveld A. S. Huibers M. M. H. Vink A. (2019). Potential of Mesenchymal- and Cardiac Progenitor Cells for Therapeutic Targeting of B-Cells and Antibody Responses in End-Stage Heart Failure. PLoS One 14, e0227283. 10.1371/journal.pone.0227283 31891633 PMC6938331

[B114] Van Linthout S. Savvatis K. Miteva K. Peng J. Ringe J. Warstat K. (2011). Mesenchymal Stem Cells Improve Murine Acute Coxsackievirus B3-Induced Myocarditis. Eur. Heart J. 32, 2168–2178. 10.1093/eurheartj/ehq467 21183501 PMC3164101

[B115] Wang H. Chen T. Ding T. Zhu P. Xu X. Yu L. (2011). Adipogenic Differentiation Alters the Immunoregulatory Property of Mesenchymal Stem Cells through BAFF Secretion. Hematology 16, 313–323. 10.1179/102453311x13085644679944 21902897

[B116] Wang M. Yan L. Li Q. Yang Y. Turrentine M. March K. (2020). Mesenchymal Stem Cell Secretions Improve Donor Heart Function Following *Ex Vivo* Cold Storage. J. Thorac. Cardiovasc. Surg. 10.1016/j.jtcvs.2020.08.095 PMC7921217 32981709

[B117] Wang S.-s. Hu S.-w. Zhang Q.-h. Xia A.-x. Jiang Z.-x. Chen X.-m. (2015). Mesenchymal Stem Cells Stabilize Atherosclerotic Vulnerable Plaque by Anti-inflammatory Properties. PLoS One 10, e0136026. 10.1371/journal.pone.0136026 26288013 PMC4546153

[B118] Wang S. Zhu R. Li H. Li J. Han Q. Zhao R. C. (2019). Mesenchymal Stem Cells and Immune Disorders: from Basic Science to Clinical Transition. Front. Med. 13, 138–151. 10.1007/s11684-018-0627-y 30062557

[B119] Wang X. Lazorchak A. S. Song L. Li E. Zhang Z. Jiang B. (2016b). Immune Modulatory Mesenchymal Stem Cells Derived from Human Embryonic Stem Cells through a Trophoblast‐like Stage. Stem Cells 34, 380–391. 10.1002/stem.2242 26523849

[B120] Wang Y.-C. Ma H.-D. Yin X.-Y. Wang Y.-H. Liu Q.-Z. Yang J.-B. (2016a). Forkhead Box O1 Regulates Macrophage Polarization Following *Staphylococcus aureus* Infection: Experimental Murine Data and Review of the Literature. Clinic Rev. Allerg Immunol. 51, 353–369. 10.1007/s12016-016-8531-1 26924010

[B121] Wang Z. Zhao Y. (2018). Gut Microbiota Derived Metabolites in Cardiovascular Health and Disease. Protein Cell 9, 416–431. 10.1007/s13238-018-0549-0 29725935 PMC5960473

[B122] Wei Z. Qiao S. Zhao J. Liu Y. Li Q. Wei Z. (2019). miRNA-181a Over-expression in Mesenchymal Stem Cell-Derived Exosomes Influenced Inflammatory Response after Myocardial Ischemia-Reperfusion Injury. Life Sci. 232, 116632. 10.1016/j.lfs.2019.116632 31278944

[B123] Wing J. B. Tanaka A. Sakaguchi S. (2019). Human FOXP3+ Regulatory T Cell Heterogeneity and Function in Autoimmunity and Cancer. Immunity 50, 302–316. 10.1016/j.immuni.2019.01.020 30784578

[B124] Wynn T. A. Chawla A. Pollard J. W. (2013). Macrophage Biology in Development, Homeostasis and Disease. Nature 496, 445–455. 10.1038/nature12034 23619691 PMC3725458

[B125] Xu R. Zhang F. Chai R. Zhou W. Hu M. Liu B. (2019). Exosomes Derived from Pro‐inflammatory Bone Marrow‐derived Mesenchymal Stem Cells Reduce Inflammation and Myocardial Injury via Mediating Macrophage Polarization. J. Cel Mol Med 23, 7617–7631. 10.1111/jcmm.14635 PMC6815833 31557396

[B126] Yang F. Y. Chen R. Zhang X. Huang B. Tsang L. L. Li X. (2018). Preconditioning Enhances the Therapeutic Effects of Mesenchymal Stem Cells on Colitis through PGE2-Mediated T-Cell Modulation. Cel Transpl. 27, 1352–1367. 10.1177/0963689718780304 PMC6168994 30095002

[B127] Yen B. L. Yen M. L. Wang L. T. Liu K. J. Sytwu H. K. (2020). Current Status of Mesenchymal Stem Cell Therapy for Immune/inflammatory Lung Disorders: Gleaning Insights for Possible Use in COVID ‐19. Stem Cell Transl Med 9, 1163–1173. 10.1002/sctm.20-0186 PMC7300965 32526079

[B128] Yoshida S. Miyagawa S. Toyofuku T. Fukushima S. Kawamura T. Kawamura A. (2020). Syngeneic Mesenchymal Stem Cells Reduce Immune Rejection after Induced Pluripotent Stem Cell-Derived Allogeneic Cardiomyocyte Transplantation. Sci. Rep. 10, 4593. 10.1038/s41598-020-58126-z 32165680 PMC7067786

[B129] Zhang N. Song Y. Huang Z. Chen J. Tan H. Yang H. (2020b). Monocyte Mimics Improve Mesenchymal Stem Cell-Derived Extracellular Vesicle Homing in a Mouse MI/RI Model. Biomaterials 255, 120168. 10.1016/j.biomaterials.2020.120168 32562944

[B130] Zhang Q. Fu L. Liang Y. Guo Z. Wang L. Ma C. (2018). Exosomes Originating from MSCs Stimulated with TGF‐β and IFN‐γ Promote Treg Differentiation. J. Cel Physiol 233, 6832–6840. 10.1002/jcp.26436 PMC13482522 29336475

[B131] Zhang Q. Iida R. Shimazu T. Kincade P. W. (2012). Replenishing B Lymphocytes in Health and Disease. Curr. Opin. Immunol. 24, 196–203. 10.1016/j.coi.2011.12.007 22236696 PMC3570822

[B132] Zhang R. Ma J. Han J. Zhang W. Ma J. (2019). Mesenchymal Stem Cell Related Therapies for Cartilage Lesions and Osteoarthritis. Am. J. Transl Res. 11, 6275–6289. 31737182 PMC6834499

[B133] Zhang Y. Ge X.-h. Guo X.-J. Guan S.-b. Li X.-m. Gu W. (2017). Bone Marrow Mesenchymal Stem Cells Inhibit the Function of Dendritic Cells by Secreting Galectin-1. Biomed. Res. Int. 2017, 1–19. 10.1155/2017/3248605 PMC5497648 28713822

[B134] Zhang Z. Tian H. Yang C. Liu J. Zhang H. Wang J. (2020a). Mesenchymal Stem Cells Promote the Resolution of Cardiac Inflammation after Ischemia Reperfusion via Enhancing Efferocytosis of Neutrophils. Jaha 9, e014397. 10.1161/jaha.119.014397 32079474 PMC7335576

[B135] Zhao J. Li X. Hu J. Chen F. Qiao S. Sun X. (2019). Mesenchymal Stromal Cell-Derived Exosomes Attenuate Myocardial Ischaemia-Reperfusion Injury through miR-182-Regulated Macrophage Polarization. Cardiovasc. Res. 115, 1205–1216. 10.1093/cvr/cvz040 30753344 PMC6529919

[B136] Zhou C. Wu X.-R. Liu H.-S. Liu X.-H. Liu G.-H. Zheng X.-B. (2020). Immunomodulatory Effect of Urine-Derived Stem Cells on Inflammatory Bowel Diseases via Downregulating Th1/Th17 Immune Responses in a PGE2-dependent Manner. J. Crohns Colitis 14, 654–668. 10.1093/ecco-jcc/jjz200 31841595

